# Promoting Peer Connectedness Through Social-Emotional Learning: Evaluating the Intervention Effect Mechanisms and Implementation Factors of a Social-Emotional Learning Programme for 9 to 12-Year-Olds

**DOI:** 10.1007/s10964-023-01871-x

**Published:** 2023-10-05

**Authors:** Isabella Pollak, Katharina A. M. Stiehl, James Birchwood, Beate Schrank, Kerstin Angelika Zechner, Christian Wiesner, Kate Anne Woodcock

**Affiliations:** 1 D.O.T. Research Group for Mental Health of Children and Adolescents, Ludwig Boltzmann Society at Karl Landsteiner University of Health Sciences, Krems, Austria; 2 Karl Landsteiner University of Health Sciences, Scientific Working Group, D.O.T.—Die offene Tür (The open door), Krems, Austria; 3 Centre for Applied Psychology, School of Psychology, University of Birmingham, Birmingham, UK; 4 Department of Health and Clinical Psychology, University of Vienna, Vienna, Austria; 5 Karl Landsteiner University of Health Sciences, Research Centre Transitional Psychiatry at the Tulln University Hospital, Krems, Austria; 6 School of Education, University of Birmingham, Birmingham, UK; 7 Department of Psychiatry, University Hospital Tulln, Tulln an der Donau, Austria; 8 Department of Diversity, University College of Teacher Education in Lower Austria, Baden, Austria; 9 Institute for Mental Health, School of Psychology, University of Birmingham, Birmingham, UK

**Keywords:** Peer relationships, Peer connectedness, Early adolescence, Social-emotional learning, Intervention

## Abstract

There is little evidence regarding the effect mechanisms of social-emotional learning programs on children’s peer relationships. The current study evaluated a novel school-based social-emotional learning program for the first year of secondary school assessing effects on social-emotional skills, peer connectedness, happiness, student and teacher classroom climate. The sample included 19 intervention classrooms (*n* = 399) and 16 waitlist-control classrooms (*n* = 281), with a mean age of 10.34 (*SD* = 0.76) and 48.8% girls. The main intervention effect analysis followed a per-protocol approach and was thus conducted with eight classes that finished all sessions (*n* = 195) and the control group classes (*n* = 281). It was further hypothesized that increases in social-emotional skills would predict peer connectedness and class climate increases, which would predict happiness. Results indicated significant intervention effects for social skills, peer connectedness and happiness. Classroom climate declined for both groups, seemingly affected by the school transition. Hypothesized relationships between target variables were partly supported with significant effects of social-emotional skills on connectedness and significant effects of peer connectedness on happiness for children reporting connectedness decreases. Additional analyses were conducted including all classrooms to compare the intervention’s effectiveness across different implementation progress groups. Significant group differences were found, indicating that implementation aspects impact intervention outcomes. The findings indicate that universal, school-based social-emotional leaning programs are effective approaches to support peer relationships in the context of the school transition. However, more implementation support seems to be needed to ensure best-practice delivery and achieve maximal intervention effectiveness.

## Introduction

Fostering children’s emotional wellbeing and social-emotional skills has become an increasing priority of schools, as emphasized in educational policy documents (Ofsted, 2019; NICE, 2008) and by the inclusion of student wellbeing measures in the Program for International Student Assessment (PISA) in addition to its traditional academic measures (Borgonovi & Pal, 2016). With 12% of students across Organization for Economic Cooperation and Development (OECD) countries reporting compromised happiness and decreased feelings of school belonging (OECD, 2017), promoting student wellbeing is a timely topic. Existing universal, preventive programs for children – including social-emotional learning (SEL) (Blank et al., 2008; Mackenzie & Williams, 2018), positive psychology (Tejada-Gallardo et al., 2020) or positive youth development (PYD) programs (Taylor et al., 2017) – have been found to commonly address two comprehensive positive development characteristics: (1) management of emotions and related behaviors and (2) positive engagement with others. In other words, improving social-emotional skills and peer relationships is at the heart of most current childhood prevention approaches. While a considerable amount of literature focuses on social-emotional learning (SEL) programs (Durlak et al., 2011, 2022; van de Sande et al., 2019), peer relationship programs have not received the same attention (Pollak et al., 2022). Additionally, there is a lack of empirical studies on the relationship between social-emotional skills outcomes and peer relationship outcomes of universal, preventive programs. Therefore, this study aimed to address this research gap by presenting a novel social-emotional learning program for the classroom context and evaluating its effects on social-emotional skills, peer relationships, classroom climate and happiness as well as the relationship between these outcome variables.

### Existing Evidence Regarding the Relationship Between Social-Emotional Skills and Peer Relationship Outcomes of Intervention Programs

Programs focusing on social-emotional skills have been found to successfully improve social skills with medium to large effects (Gutman & Schoon, 2015). As prosocial behavior is vital for establishing friendships (Bowker et al., 2010), social-emotional learning programs have been discussed as a means to facilitate peer relationships (Brendgen & Poulin, 2018; Suldo et al., 2013). Indeed, there is promising evidence regarding long-term effects of PYD interventions and school-based SEL programs on peer relationships (Pollak et al., 2022; Taylor et al., 2017). A review of programs aiming to facilitate peer relationships (Pollak et al., 2022) found preventive, universal, school-based programs to positively affect peer relationships through positive psychology (Shoshani et al., 2016) classroom interactions (Mikami et al., 2005), mindfulness (Lombas et al., 2019; Schonert-Reichl et al., 2015; Terjestam et al., 2016) and emotional resilience (Kozina, 2020; Maalouf et al., 2020; Siu, 2007; Stallard et al., 2007). Typical social-emotional skills programs, however, commonly addressed neurodiverse and at-risk populations (Pollak et al., 2022), even though social-emotional learning is promoted as the ideal universal, preventive approach in schools (Greenberg et al., 2017). Thus, there is a lack of empirical studies addressing the impact of universal, preventive social-emotional learning approaches in school on peer relationships.

Furthermore, many evaluation studies do not collect outcome measures on all of the skills they address (van de Sande et al., 2019) and evidence from longitudinal or mediation studies is rare (Gutman & Schoon, 2015). Thus, there is a need for more studies to address effect mechanisms initiated by interventions (Domitrovich et al., 2017). Results from the few intervention studies assessing relationships between social-emotional skills and peer relationship outcomes, suggest an impact of prosocial behavior interventions on peers’ perception of classmates (Palacios et al., 2019) and an impact of peer relationships on behavior outcomes of intervention programs (Witvliet et al., 2009). However, a longitudinal intervention study did not find effects of target skills on loneliness outcomes (Orkibi et al., 2017). Thus, it remains largely unclear how improvements in social-emotional skills through universal prevention programs impact peer relationships in the classroom-context.

Additionally, age-related and school-year-specific context factors seem to form a crucial background for the relationship of social-emotional skills and peer relationships. School-based programs seem most effective during developmental transition periods, such early adolescence (January et al., 2011; Mackenzie & Williams, 2018). During early adolescence, brain regions related to emotions are especially malleable, which makes this period ideal for social-emotional interventions (Jansen & Kiefer, 2020). At the same time, peers have been found to impact social behaviors bi-directionally during adolescence (Orson et al., 2020), as this is a period of heightened focus on group norms (Hazen et al., 2008). Particularly the social-emotional environment in the classroom and classroom interactions have been found to impact individual student behavior (Busching & Krahé, 2020; Wang et al., 2020), although related evidence is still inconsistent (Wang et al., 2020). Overall, positive peer relationships in the classroom have been linked to positive classroom environments (Khalfaoui et al., 2021), and positive school climate has been found to be a major predictor of student’s life satisfaction (Suldo et al., 2013).

However, the primary-secondary school transition – which falls in the period of early adolescence – can be difficult for some children (van Rens et al., 2018) and causes particular instability regarding peer relationships (Ng-Knight et al., 2019). While wellbeing and school climate in primary school are high, there is a decrease after the transition to secondary school in various European countries (Coelho et al., 2020; Eder, 2007; Tobia et al., 2019). Maintaining close friends over the transition was, however, associated with positive mental health aspects (Ng-Knight et al., 2019) and happiness at school (Heinsch et al., 2020). Social-emotional skills seem to play a crucial role in this context, as being perceived as prosocial was found to be essential for friendship formation over the elementary-secondary school transition (Bowker et al., 2010). However, associations between wellbeing at school and the relationship to school friends are bi-directional with a suggested moderating role of emotions (Kiuru et al., 2020). Thus, intervention efforts to facilitate students’ social-emotional wellbeing and their management of peer relationships were suggested to support students during the transition period (Bagnall et al., 2020). However, to fully understand the role of social-emotional learning in the classroom, and its impact on peer relationships and happiness in the context of the school transition, their intervention effect mechanisms, i.e. the relationship between these intervention target variables, need to be assessed (Domitrovich et al., 2017).

### Implementation Factors

A need to address implementation aspects in an educational setting is emphasized by researchers (Green et al., 2021) and policy documents (Borgonovi & Pal, 2016; Kankaraš et al., 2019). While the integration of universal, preventive social-emotional skills programs in school practices has been suggested due to cost-effectiveness and sustainability advantages (Domitrovich et al., 2017), intervention effectiveness largely depends on high quality implementation (Durlak & DuPre, 2008). Specifically complex interventions, which address multiple interacting components – such as social-emotional learning programs – come with implementation and evaluation challenges relating to standardization, context sensitivity, and the complexity of the effect model of intervention outcomes (Craig et al., 2019). Thus, to increase implementation quality, two aspects need special consideration; (i) a well-defined intervention effect model and (ii) a “support system” providing infrastructure and training elements to create a common delivery context and decrease variability of implementation quality (Domitrovich et al., 2008).

As the intervention model should feature core elements derived from theory and evidence-base, (Domitrovich et al., 2008), a model of social-emotional skills increases and their associations to outcome variables such as peer relationships and wellbeing was developed. Additionally, standardization and delivery aspects should be considered (Domitrovich et al., 2008). Thus, evidence regarding timing and appropriate delivery methods (January et al., 2011; Pollak et al., 2022) was reviewed. To measure implementation fidelity of the delivery of this intervention model, adherence is fundamental (Carroll et al., 2007).

The support system should be tailored to the intervention deliverers. While universal school-based interventions have been found to be more effective when delivered by school-staff as compared to external trainers (Durlak et al., 2011), teachers lack professional development opportunities and feel ill-equipped to address social-emotional or mental health needs (Bale et al., 2020; Martínez, 2016). Implementation staff’s self-efficacy seems to interact with intervention complexity in regards to student outcomes (Caron et al., 2020), emphasizing the importance of targeted support systems in complex interventions. However, a support system merely aids the implementation and needs to be understood as distinct from intervention strategies (Domitrovich et al., 2008). A teacher training alone, for example, did not increase students’ peer relationships (Mikami et al., 2011). It is further unclear whether changing teachers’ attitudes even impacts their behaviors in class, with some studies supporting such a relation (Wilson et al., 2022), while others do not (Garrote et al., 2020). However, a combination of personal teacher effects and classroom setting effects were found to impact intervention results (Tolan et al., 2020). Thus, intervention complexity, facilitation strategies, quality of delivery, and participant responsiveness were identified as moderators of implementation fidelity (Carroll et al., 2007).

### A Novel Social-Emotional Learning Program and its Intervention Effect Model

Based on this evidence-base regarding associations between social-emotional skills, peer connectedness and happiness in school, a novel prevention program was developed to support children after the transition from primary to secondary school in the Austrian school context. The central assumption was that social-emotional skills can be taught in social-emotional support programs (Gutman & Schoon, 2015), which would have consequential effects on students’ peer relationships and happiness (Bowker et al., 2010; Flannery & Smith, 2017; Heinsch et al., 2020) – while relationships and happiness cannot be taught directly. Specifically, the program aimed to train emotional competencies and prosocial behaviors relevant for peer relationships (Flannery & Smith, 2017), assuming that high interpersonal skills will lead to successful interpersonal interactions (Tolan et al., 2016) increasing peer connectedness in the classroom (Bowker et al., 2010) and student classroom climate (Khalfaoui et al., 2021). In turn, by supporting peer relationships in class after the school transition, the program aimed to facilitate children’s happiness in class (Heinsch et al., 2020).

Based on the Medical Research Council (MRC) guidance for intervention development and evaluation (Skivington et al., 2021), the described relationships between target variables (social-emotional skills, peer connectedness, class climate, happiness) were used to develop an intervention effect model in order to (a) identify potential weak links in the causal chain and (b) identify whether such a weak link may be the cause of ineffectiveness on higher outcome levels (e.g., happiness). As mediation analyses would be the gold standard to fully understand the effect mechanisms of social-emotional learning in the classroom (Domitrovich et al., 2017), this study tried to assess requirements that could inform future mediation studies and inform theory regarding how to intervene (Skivington et al., 2021). First, this study aimed to evaluate intervention outcomes and their effect sizes to establish that the intervention in question actually affected the variables of interest and secondly it assessed relationships between changes in the target variables to establish correlations which are the basis of mediation analyses.

Early adolescence was chosen as target age period, as social awareness and receptiveness for social input is particularly high during this period (Hazen et al., 2008) and it was found to be a particularly promising time for implementing social-emotional learning (Jansen & Kiefer, 2020; January et al., 2011). Specifically, the period of school transition was chosen to buffer against potential difficulties with the transition itself (van Rens et al., 2018), and establish favorable classroom norms (Chang, 2004). The first year of secondary school has been suggested as an ideal opportunity to facilitate students’ adaptation to the new classroom context and promote their wellbeing (Rice et al., 2015). With the primary-secondary school transition in the Austrian context in mind, the program targeted children aged 9 to 12.

## Current Study

While universal, school-based social-emotional learning programs have received much attention in recent years, there is limited evidence regarding their effect mechanisms on peer relationships and happiness. This study had two main aims; firstly, it aimed to assess the intervention effects of a novel social-emotional learning program for the Austrian primary-secondary school transition context on target variables social-emotional skills, peer connectedness, student generated class climate (i.e., students’ reports of positive interactions in class), teacher generated class climate (i.e. students’ reports on the teacher’s encouragement of positive interactions in class) and happiness in class. Secondly, this study aimed to explore the relationship between the intervention effects of these target variables to address a gap in knowledge concerning the intervention effect mechanisms of social-emotional skills programs on peer connectedness. In line with these aims, it was hypothesized that compared to a waitlist control group, the intervention group will report increased social-emotional skills over the intervention period (T1-T2) (hypothesis 1). It was further hypothesized that compared to the control group, the intervention group will report increased peer connectedness, happiness and classroom responsibility climate, both that generated by teachers and that by classmates, over the study period (T1-T3) and over the follow-up period (T2-T3) (hypothesis 2). To explore relationships between these outcomes – and potentially prepare for future mediation studies – an intervention effect model was developed, which predicted that, *in the intervention group*, immediate intervention effects on social-emotional skills during the intervention period (T1-T2 changes) would predict classroom climate generated by students and peer connectedness after the intervention period (T2-T3 changes) (hypothesis 3). It was further expected that, *in the intervention group*, peer connectedness changes (T2-T3) would correlate with happiness changes (T2-T3) (hypothesis 4). Additionally, exploratory analyses concerning the potential impact of implementation fidelity on outcome variables were conducted. In line with a framework on evaluating implementation fidelity (Carroll et al., 2007), it was first explored, whether higher adherence to the program manual would result in better outcomes regarding social-emotional skills, class climate, connectedness and happiness. Secondly, moderating factors were assessed. It was explored whether teachers reporting more perceived relevance of contents were also perceived as encouraging positive class climate by their students (teacher generated class climate). Lastly, it was tested whether students’ perceived teacher generated class climate (i.e., how much the teacher encourages positive behavior in class) predicted student generated class climate (i.e., their report of actual behavior in class) (Saarento et al., 2015).

## Methods

### The Program “You, Me and the Little Monsters”

“You, Me and the Little Monsters” is a social-emotional learning program for the classroom context aiming to increase class climate and further peer connectedness among students. Eight consecutive sessions introduced students to their “emotion monsters”, which are neither good nor bad but always accompany people in their everyday lives. Emotional vocabulary was expanded through discussing different monster drawings and the concept of emotion regulation was introduced as trying to shrink monsters when they become too big. The discussion of topics such as empathy, conflict resolution, and bullying was supported by short digital stories, that presented children with characters in a classroom and allowed children to drive the storyline by asking them how the characters should react to the situations. By discussing authentic scenarios of school-life and putting newly developed classroom rules into practice, the program aimed to facilitate social-emotional skills and thereby increase classroom climate and peer connectedness. An overview of the content delivered in each session can be found in Table 1. Programme materials can be found at https://osf.io/v59eg/.

**Table 1:** Overview of intervention sessions

Session	Target competencies	Description of the session	Methods used during the session
1	Emotion vocabulary, recognising emotions in oneself and others	The metaphor of emotion-monsters is used to externalise emotions, increase emotion-vocabulary and discuss where and how we feel our emotions. Students draw their personal “happy monster” and “anger monster”.	Group discussion of monster cartoon prompts, drawing exercise, games, workbook exercise
2	Rating the intensity of emotions, emotion regulation	With the metaphor of small, medium, big and huge emotion-monsters, students learn to rate the intensity of their emotions. Emotion regulation strategies are introduced as ways to shrink monsters. In Joe’s story, emotion regulation strategies are discussed.	Group discussion & poster activity, workbook exercise, mindfulness, story
3	Classroom rules, empathy and perspective taking	Students decide on their own classroom rules to feel welcome and safe in their class. Joe’s story highlights the importance of prosocial behavior in class and serves as example to reflect on others’ feelings, thereby practicing empathy and perspective taking.	Group dynamic games, group discussion & post-it brainstorming, story
4	Empathy, interpersonal emotion regulation	The classroom rules are applied in stories with specific examples to practice prosocial behaviors. Empathy and strategies to support classmates are practiced.	Cooperation game, group reflection, story, individual reflection
5	Communication skills, conflict resolution	Students learn to rate problems and decide whether they are (i) minor and will resolve themselves, (ii) medium and need addressing, or (iii) big and require adult help. In Ana’s story, communication is introduced as first and most important step to resolve conflicts.	Circle time to reflect on class community, games, story, partner activity, mindfulness
6	Conflict resolution strategies	In specific scenarios, students try to take different characters’ perspectives. Conflict resolution strategies are introduced and discussed in Ana’s story.	Circle time, partner activity, workbook exercise, story
7	Bullying, bystander-effect, support strategies	Students learn to treat bullying as a big problem that requires help to solve. The bystander-effect is discussed to encourage all students to support each other when witnessing bullying behavior.	Circle time, workbook exercise, story
8	Recap of emotion regulation, prosocial behavior strategies, conflict resolution	Through a story about the “anger-monster” that is passed on from student to student, children recap learned strategies to regulate their emotions, support each other and resolve conflicts.	Circle time, workbook exercise, story, reflection

The program is based on empirical evidence. Specifically, understanding emotions, emotional support and prosocial behaviors were found to be important for friendship quality and peer liking, while conflict management seems important for friendship stability (Flannery & Smith, 2017). Classroom prosocial behavior specifically, was found to predict individual social behavior (Busching & Krahé, 2020) and social-emotional wellbeing (Wang et al., 2020). Additionally, how students perceived their teachers’ attitudes predicted self-reported behavior and mediated intervention outcomes (Saarento et al., 2015). Thus, school-based intervention efforts were advised to combine a focus on skill instruction and school climate (Domitrovich et al., 2017), which was implemented in this program by addressing emotion regulation, empathy and conflict management skills alongside a focus on positive classroom management techniques. The program activities further reflected the underlying principles of Positive Psychology (Seligman, 2002) by focusing and strengths and skills as well as Vygotsky’s and Bandura’s learning theories (Bandura, 1977; Vygotsky, 1978), by making the social (classroom) context central to all skills training tasks. In line with this, important concepts, such as emotion regulation, were expected to require some form of modeling (e.g., in stories) and interaction with peers.

Additionally, this program considered empirical evidence and theoretical frameworks concerning the school-transition. The program was designed to address a potential mismatch between children’s transition worries and their emotional skills to cope with these worries (Bagnall et al., 2021), while appreciating that children can be simultaneously excited and worried about novel experiences (Jindal-Snape & Cantali, 2019). This notion is linked to the stage-environmental-fit theory’s proposition that decreases in motivation and behavior changes in early secondary school might be related to insufficient support and preparation in the school context (Eccles, et al. 1997). Thus, the multiple and multi-dimensional transitions theory (Jindal-Snape & Rienties, 2016) was considered, which emphasizes that children experience multiple transitions simultaneously in various domains. Therefore, the program focused on emotional aspects (e.g., communicating emotions, emotion regulation), social aspects (e.g., making new friends) as well as educational aspects (e.g., suitable classroom atmosphere, classroom rules).

The program was developed by a group of psychologists, psychiatrists and pedagogists from the University of Birmingham, UK, the Karl Landsteiner University of Health Sciences, Austria, the Ludwig Boltzmann Society, Austria, the University of Vienna, Austria as well as the University College of Teacher Education in Lower Austria. It was iteratively refined during a participatory development process, following intervention development guidelines (Craig et al., 2019; Hagen et al., 2012; Hawkins et al., 2017; Skivington et al., 2021). Multiple schools were involved in this development process over the span of three years, with students of the target age group consistently providing input concerning appropriate and relevant program contents and activities and teachers reviewing practicality and feasibility aspects. For examples of qualitative studies conducted as part of this development effort see Krammer et al., (2023), Stiehl et al., (2023), and Pollak et al. (in press).

### Study Design

This study followed a clustered waitlist-control evaluation design with intervention and control group and three measurement points at pre, post and follow-up. Participants were clustered in their classrooms due to the teacher-lead implementation in schools. In spring and summer 2021, 44 classes were contacted via the research group’s existing network and recruited to participate in the study. The study was conducted between September 2021 and February 2022. While all schools and classrooms were recruited based on their motivation to implement a novel social-emotional learning program, some schools expressed concerns regarding a program start in September due to school-specific procedures at the start of the term (e.g., school trips, project weeks), and school-specific Covid-19 testing procedures, which were expected to be more frequent and time consuming in the early school months. However, a program start in September (or early October at the latest) was vital to assess realistic program benefits, as the program was intended to support children right after the transition. Therefore, the decision was made to assign those schools motivated to participate but concerned about a start in September to the control group. Thus, intervention and control group were not assigned at random. Out of 44 recruited classrooms, 23 classes were assigned to the control group.

Ethical approval for this study was obtained at Karl Landsteiner University of Health Sciences, Austria (EK-Nr. 1021/2020). Additionally, approval of the Lower Austrian school authorities was obtained. Schools were recruited through an existing network of the research group and a cooperation with the Lower Austrian Teacher Education University. Headteachers and class teachers were contacted by telephone and received detailed information about the program and the study. When class teachers agreed to piloting the program, adult and child-friendly information sheets were sent to teachers to be read with their students and forwarded to parents. Due to schools’ autonomy, teachers and school authorities decided whether to add the program to their curriculum, however parents and students decided whether to participate in the evaluation of the program. Thus, children who decided not to participate in this evaluation study were not separated from their classroom while the program was implemented, which was in line with the program’s intentions to comprehensively increase class cohesion and connectedness between all students. Written informed consent was obtained by teachers and parents and sent to the research group through the teachers. Additionally, students were shown a video recorded by the researchers (first and second author) informing them about the study aims and explaining the questionnaires and anonymity of the data. Student assent was obtained through ticking a box at the beginning of the evaluation questionnaires. Teachers were asked to provide written consent before completing fidelity measures.

Data was collected via the online questionnaire platform socisurvey via a server at University of Vienna and was later stored on a secure server at Karl Landsteiner University of Health Sciences. Students were asked to provide a self-generated personal identification code, thus, it was not possible to identify individual students and the data was anonymous. This code was used to link students’ pre, post and follow-up data. Children were instructed to generate their own code by providing the day of their birthday (e.g., 24), the first two letters of their mother’s name (e.g., LI), the number of older brothers (e.g., 0), and the number of older sisters (e.g., 1). To ensure children could easily generate the same code at all assessment points, detailed instructions and an example were provided on each questionnaire and teachers were asked to show students a video of two researchers explaining the generation of the code before each assessment point. Additionally, classroom codes were generated by the researchers to pre-face each student code and anonymize teachers’ qualitative fidelity feedback.

Intervention group teachers received a detailed program manual and were required to participate in two training sessions led by the researchers (first and second author) prior to the start of the implementation to increase implementation fidelity. Control group teachers received the same manual after finishing the follow-up questionnaire with their classrooms and were invited to voluntary training sessions. Researchers contacted intervention group teachers regularly by email and telephone to answer any questions and check for fidelity and implementation progress. Intervention group teachers were asked to complete additional written fidelity checklists for each session and both, control and intervention group teachers were asked to complete surveys concerning (additional) social-emotional lessons throughout the intervention period.

While the present paper focused on the intervention’s effects on social-emotional skills, classroom climate, connectedness and happiness, and the intervention effect mechanisms, a separate paper focused on variables concerning the impact of the school transition. Specifically, fears related to the school transition and competencies, including emotional abilities and conflict management, will be discussed with attention to gender differences in a prospective publication, which is currently in preparation.

### Participants

Out of the 21 intervention classes and 23 control classes, which were recruited to participate in the study, nine classes dropped out of the study before the completion of pre-tests in September 2021, two of which were in the intervention group and seven in the control group, all referring to Covid-19 related school measures and unforeseen workload demands. Based on teachers’ reports of the number of students in their classroom at the start of the year (which might change very slightly in the first weeks), 91.7% of children (and parents) in intervention classrooms and 94.4% of children (and parents) in control classrooms have consented to participate in this research. Pre-test measures (T1) were completed in September and October 2021 by 19 intervention classrooms (*n* = 399) and 16 control classrooms (*n* = 281). The mean age of participants was 10.34 (*SD* = 0.76) and 48.8% were girls. Out of the intervention classes, another three classes dropped out of the study after completing pre-measures, due to Covid-19 related or other school-related workload increases. Post-test measures were completed by 16 intervention and 16 control classrooms in November or December 2021. Intervention class teachers were asked to implement one session per week for eight weeks and complete the post-test soon after the last session. Depending on the number of lessons class teachers had with their class, school holidays, and Covid-19 related measures in their classrooms, the actual implementation period varied between 5 to 13 weeks in intervention classes, with a mean of 10 weeks. Similarly, for control classrooms the period between pre and post measures varied between 5 and 12 weeks, with a mean of 9.8 weeks. Aiming for a minimum of a six-week follow-up period, teachers were asked to complete the follow-up measures (T3) in late January or February 2022. Follow-up measures (T3) were obtained from 29 of the remaining classrooms (*n* = 486), including 14 intervention classes and 15 control classes. The follow-up period ranged between 5 and 14 weeks (mean of 8.8 weeks) for intervention classes and 4 to 8 weeks for control classes (mean of 6.8 weeks).

Out of the intervention group, eight classes did not implement all eight program sessions due to time constraints, Covid-19 related issues (additional time spent on testing children for Covid-19, illness etc.) or other school-related commitments. Six classes completed at least six sessions (“partly finished”, *n* = 116), two classes completed less than five sessions (“few sessions”, *n* = 42) and three classes dropped out of the study completely (“drop-out”, *n* = 46). Following the MRC guidance’ differentiation of efficacy of the intervention in “ideal” circumstances and the effectiveness in “real-world” settings (Skivington et al., 2021), this study first explored the intervention’s efficacy when all sessions were implemented to assess whether the intervention itself has the potential to achieve intended outcomes. Thus, the main intervention effect analysis followed a per-protocol approach, including only the eight classes that finished all sessions as described in the manual (*n* = 195) and the control group classes with pre and post measures (*n* = 281). In a second step, all classes were included in a separate analysis on the impact of implementation fidelity on intervention outcomes. This analysis compared the effectiveness of the intervention in different implementation progress stages (e.g., when few sessions only were completed).

In the eight classes that finished all sessions, there was missing data for 19 students, eight of whom only completed pre-measures, and eleven of whom completed two out of three measurement-rounds. In the control group, there was missing data for 75 students, 24 of whom completed pre-measures, and 51 of whom completed two out of three measurement-rounds. Demographic characteristics of the groups included in the main intervention effect analysis can be found in Table 2. The share of school type^1^ was almost even within groups and equal across groups in the initially recruited sample, with 11 grammar school classes out of 21 intervention classes (52.4%) and 11 grammar school classes out of 23 control classes (47.8%). However, more comprehensive schools dropped out in the intervention group and more grammar school dropped out in the control group, leaving the sample quite unbalanced in this regard.

**Table 2:** Demographic information of the control group and “full programme” intervention group

		Control group		Intervention group	
		Count	Percentage	Count	Percentage
Participants	N	281	59%	195	40.9%
Age (years)	9	1	0.3%	1	0.5%
	10	175	66.3%	148	82.2%
	11	82	31.1%	29	16.1%
	12	6	2.3%	2	1%
	n/a	17		15	
Gender	girl	133	50.4%	88	48.9%
	boy	131	49.6%	92	51.1%
	n/a	17		15	
School Type	grammar school	104	37.1%	170	87.2%
	comprehensive school	177	62.9%	25	12.8%
School Setting	rural	96	34.2%	94	48.2%
	urban	185	65.8%	101	51.8%

Demographic data is provided for the control classrooms and intervention classrooms, which finished all programme sessions and were included in the main analysis. Percentage of N compares distribution of the whole sample across the intervention and control group, while percentage data in all other categories indicates the percentage of the subgroup (e.g. girls) within the respective group (e.g. gender in control group). N/A indicates that this information was not provided by participants, N/A was not considered when calculating percentage points. Data was collected at pre-test

### Measures

#### Happiness

Happiness was measured with the subscale “happiness” of the EPOCH Measure of Adolescent Wellbeing (Kern et al., 2016). The scale was translated from English to German and adapted to the school context and validated with 10 to 18-year-olds (Buerger et al., 2023). It was chosen for its brief, targeted items focused on the school environment (e.g., “I’m mostly happy when I’m at school”). The subscale consisted of four items with a five-point Likert scale ranging from 1-“not true at all” to 5-“very true”. In the original measure, the happiness subscale had an internal consistency of α = 0.83, and re-test reliability of *r* = 0.49 (Kern et al., 2016). In the current study, the happiness subscale had an internal consistency of α = 0.66.

#### Peer connectedness

Peer connectedness was measured with the subscale “peer connectedness” of the EPOCH Measure of Adolescent Wellbeing (Kern et al., 2016), which had a similar focus on the school environment as the happiness subscale. The subscale consisted of four items with a five-point Likert scale ranging from 1-“not true at all” to 5-“very true” (sample item; “I have friends in school, who are really important to me”). In the original measure, the connectedness subscale had an internal consistency of α = 0.77, and re-test reliability of *r* = 0.42 (Kern et al., 2016). In the current study, the connectedness subscale had an internal consistency of α = 0.80.

#### Social-emotional skills

Social-emotional skills were measured with one subscale of the Self-Report Checklist for Social and Learning Behavior (Lohbeck et al., 2014). As the learning behavior related subscales (cooperation in group-work, concentration, self-efficacy, perseverance, autonomy) were not of interest, and the remaining subscales focused on self-centered skills (self-control, self-assertion, self-awareness) or peer relationships directly (social contact with peers), the subscale empathy / empathic concern was used. The subscale comprises of four items, referring to empathic, prosocial behaviors in a classroom setting, including “I comfort classmates after negative experiences”, “I encourage classmates, when they are sad”. This subscale has strong correlations with the SDQ subscales prosocial behavior and emotional problems (Lohbeck et al., 2014), while the use of the SDQ itself has been discouraged due to its limited change sensitivity (Unwin et al., 2018). Additionally, the development of many social-emotional skills and their importance for friendships seem to differ between boys and girls (Flannery & Smith, 2017; Ross et al., 2019), while prosocial behaviors have been found to be important for all children’s friendships (Flannery & Smith, 2017). Thus, this subscale was chosen to measure empathic, prosocial behaviors as intervention outcome hypothesized to be relevant for all children’s peer relationships. Students rated themselves on a four-point Likert scale from 0-“never”, 1-“rarely”, 2-“sometimes” to 3-“regularly”. The questionnaire was developed and validated in German, with 9 to 19-year-old youth. In the validation research, the subscale had an internal consistency of Cronbach α = 0.83 and a re-test reliability of *r* = 0.67 (Lohbeck et al., 2014). In the current study, the scale had an internal consistency of α = 0.79.

#### Student and Teacher Generated Classroom Climate

Student and teacher classroom climate were measured with the student and teacher subscales of the classroom responsibility climate questionnaire (Fernández-Río et al., 2019). This measure was chosen because it focused on the classmates’ and teachers’ behavior contributing to the classroom dynamic, which was the focus of this intervention. Both subscales had 5 items (e.g., student classroom climate “During class, we encourage each other” / teacher classroom climate “The teacher likes that we encourage each other”) to be rated on a 7-point Likert scale from 1-“strongly disagree” to 7-“strongly agree”. The questionnaire was developed and validated in Spanish with secondary school students. The items were translated to German by the researchers using a parallel blind translation approach (Behling & Law, 2000). In the Spanish version, the student and teacher subscales had a measurement reliability of Cronbach α = 0.81 and α = 0.89 respectively (Fernández-Río et al., 2019). In the current study, the student class climate scale had an internal consistency of α = 0.81 and the teacher class climate scale had an internal consistency of α = 0.76.

### Fidelity Strategy

This evaluation was teacher-implemented to increase sustainability of the intervention at schools and students’ acceptability through familiar delivery personnel (Mackenzie & Williams, 2018). However, as this study was conducted in the context of heightened Covid-19 precautions in schools, the researchers were unable to be present for sessions or collect observational fidelity measures. Therefore, a detailed fidelity strategy was developed based on a fidelity framework by Carroll et al. (2007), which advises indexing implementation fidelity by measuring adherence (specifically content, coverage, duration and frequency) and its moderators (specifically complexity, delivery quality, additional facilitation strategies, participants’ responsiveness).

As for the moderating factors, more detailed and specific interventions typically achieve higher fidelity (Carroll et al., 2007). By establishing a support system for an intervention, an appropriate delivery context is created, thus decreasing the variability of the quality of implementation (Domitrovich et al., 2008). Thus, a very detailed teacher manual and student workbook were developed with additional materials such as posters and digital stories to help targeted and guided discussion of contents. Additional facilitation strategies, such as the implementation of two online teacher training workshops, regular email reminders and bi-monthly phone calls to discuss progress and any problems were implemented.

To measure the remaining fidelity aspects, a teacher fidelity questionnaire was sent out after each session. Adherence to the program manual was measured by asking teachers to report on the duration and frequency of their program implementation and report their content coverage by indicating how comprehensively they had discussed each activity on a scale from 1 to 5 (1-left out completely, 2-discussed in less detail, 3- completed, 4- discussed in more detail, 5- discussed very thoroughly). Quality of delivery itself was difficult to assess as researchers could not be present for the implementation (due to Covid-19 related measures). Instead, teachers’ perceived relevance of contents for their students was assessed because teachers’ explicit attitudes have been found to predict their behavior in class (Wilson et al., 2022). Thus, it was assumed that their perceived relevance would predict their implementation of these contents. Perceived relevance of program contents for their students was measured on a scale from 1 to 5 (1-not relevant, 2-little relevance, 3-ok, 4-relevant, 5-highly relevant). As participant responsiveness could not be directly assessed by the researchers either, teachers were asked to rate their perceived student responsiveness (“Did you feel your students understood all concepts?”) on a scale from 1 to 5 (1-didn’t understand, 2-understood little, 3-understood ok, 4-fully understood, 5-fully understood and capable of using new competencies). Additional open questions provided room to detail potential deviations of the script and reasons for such deviations.

### Statistical Analysis

The statistical analysis was conducted using IBM SPSS Version 29. Linear mixed models were computed to evaluate intervention effects (Hypotheses 1–2) and relationships between change scores (Hypotheses 3–4) due to their ability to account for fixed and random effects (Detry & Ma, 2016). Restricted maximum likelihood estimation was used to account for missing data. For the main intervention effect analyses, a per-protocol approach was followed to assess the efficacy of the intervention in the “ideal” circumstance of a fully completed program (Skivington et al., 2021). Models assessing intervention effects accounted for main effects of time (T1-T2 / T2-T3) and main effects of group (intervention/control) and interaction effects between group and time. Because the intervention period T1-T2 (social skills) was the period of interest for some variables, while the follow-up period T2-T3 (connectedness, happiness) was the period of interest for other variables, the analysis was always restricted to including the two levels of the factor time, which were of interest for the respective analysis^2^. Students’ ID and the classroom variable were included in the repeated structure of the model and the intercept of classroom was specified as a random effect. As maximum likelihood estimation was used, missing data was estimated from present data and no participants were excluded due to missing data. Effect size calculation can be problematic in multilevel models as calculating a change in residual variance when adding predictors to the model could lead to impossible values (Hox, 2013). It has thus been suggested to interpret effects sizes derived from simple methods as merely indicative (Hox, 2013). For models with factors in the fixed effects structure (e.g., group, time), partial eta squared was computed from the F-statistic, specifically the F-value and its degrees of freedom (Lakens, 2013).

As this study evaluated a novel intervention, it aimed to assess basic requirements to establish the intervention’s effect mechanisms. Thus, the present analysis assessed (a) the intervention’s effects on its target variables and (b) the relationships (i.e., correlations) between changes in these target variables (as specified in intervention effect model). Basic intervention effect mechanisms were evaluated by analyzing correlations between skills changes to understand the trajectories of targeted skills and explore requirements for future mediation studies (Gutman & Schoon, 2015). Models assessing these relationships between target variables correlated the change scores of hypothesized effect variables (e.g., social-emotional skills) with the change scores of hypothesized outcome variables (e.g., peer connectedness). The intercept of classroom was again specified in the random effects structure and maximum likelihood estimation was used. For these models, *r* was used as indicator of effect size.

Additional exploratory analyses were conducted to evaluate effects of implementation factors. To assess whether teachers’ perceptions predicted their classroom management style after the intervention (i.e., teacher generated classroom climate at T2), spearman correlation coefficient was computed. To assess whether this classroom management style (T1-T2) predicted subsequent student ratings of their classroom climate (T2-T3), a linear mixed model was computed. Additionally, the impact of adherence to the program (i.e., duration and frequency) as reflected by the implementation progress (i.e., how many sessions were completed at the end of the intervention period) on intervention outcomes was assessed. This analysis simultaneously represents an evaluation of the intervention’s effectiveness in real-world classroom contexts (Skivington et al., 2021), which might not allow for a full implementation. Intervention effects were compared between (i) eight “full program finished” classrooms finishing all sessions (*n* = 195), (ii) six “partly finished” classrooms completing at least six sessions (*n* = 116), (iii) two “few sessions” classrooms having completed less than five sessions (*n* = 42) and (iv) three classrooms dropping out of the study (*n* = 46) and (v) the 16 control classrooms (*n* = 281). When post-hoc tests were used to assess group differences, Fisher’s Least Significant Difference (LSD) test was used, as the Bonferroni method is more conservative, specifically when multiple tests are conducted (Lee & Lee, 2018). Fischer’s LSD was suggested to avoid type II error (Lee & Lee, 2018), which seemed relevant as the sample size of the different implementation groups was much smaller than the overall sample and small effects were expected.

## Results

### Fidelity to the Planned Intervention

The main indicator used to assess adherence was the completion of all eight program sessions (i.e., frequency and duration of the program), which was assessed in repeated telephone conversations. Out of 19 intervention classrooms, eight finished the full program, six “partly finished” the program, two only completed few sessions and three classes dropped out of the study. Additionally, duration and content coverage of each completed session was collected in the fidelity questionnaires.

Fidelity questionnaires were completed by 14 teachers (see Appendix 1). Teachers’ consistency in completing fidelity measures differed, with a range of completed questionnaires between 1 and 8 (one questionnaire for each of the 8 sessions). On average, teachers completed 4 questionnaires and only one teacher completed all 8 fidelity questionnaires. However, as some teachers did not implement all 8 sessions, some might have completed all questionnaires for all their completed sessions. Overall, 48.8% reported that they used one school lesson (50 min) to cover the content, while 34.4% reported having spent more time than one school lesson on one session. Teachers who finished all program sessions reported having spent an average of 59.2 min on each session, while teachers who partly finished the program spent an average of 65 min on their sessions. Teachers reported a mean of 3.05 (*SD* = 0.61) for content coverage across all sessions. With a score of 3 indicating all content was covered (and 4–5 indicating more detailed coverage of the content beyond suggested activities), this suggests, on average, teachers reported completing all suggested program activities. Teachers who finished the full program reported a mean of 2.95 (*SD* = 0.19) and teachers who partly finished the program reported a slightly higher content coverage with a mean of 3.22 (*SD* = 0.30). Regarding participants’ responsiveness, teachers reported an overall mean of 3.91 (*SD* = 0.62), indicating that, on average, teachers felt their students had achieved a good understanding of the concepts. Lastly, concerning their perceived relevance of concepts and program contents, teachers reported an average score of 4.12 (*SD* = 0.45), indicating they perceived most contents as relevant. For a detailed report of fidelity per teacher and per session see Appendix 1.

### Main Intervention Effect Models

#### Social-emotional skills

**Fig. 1:**
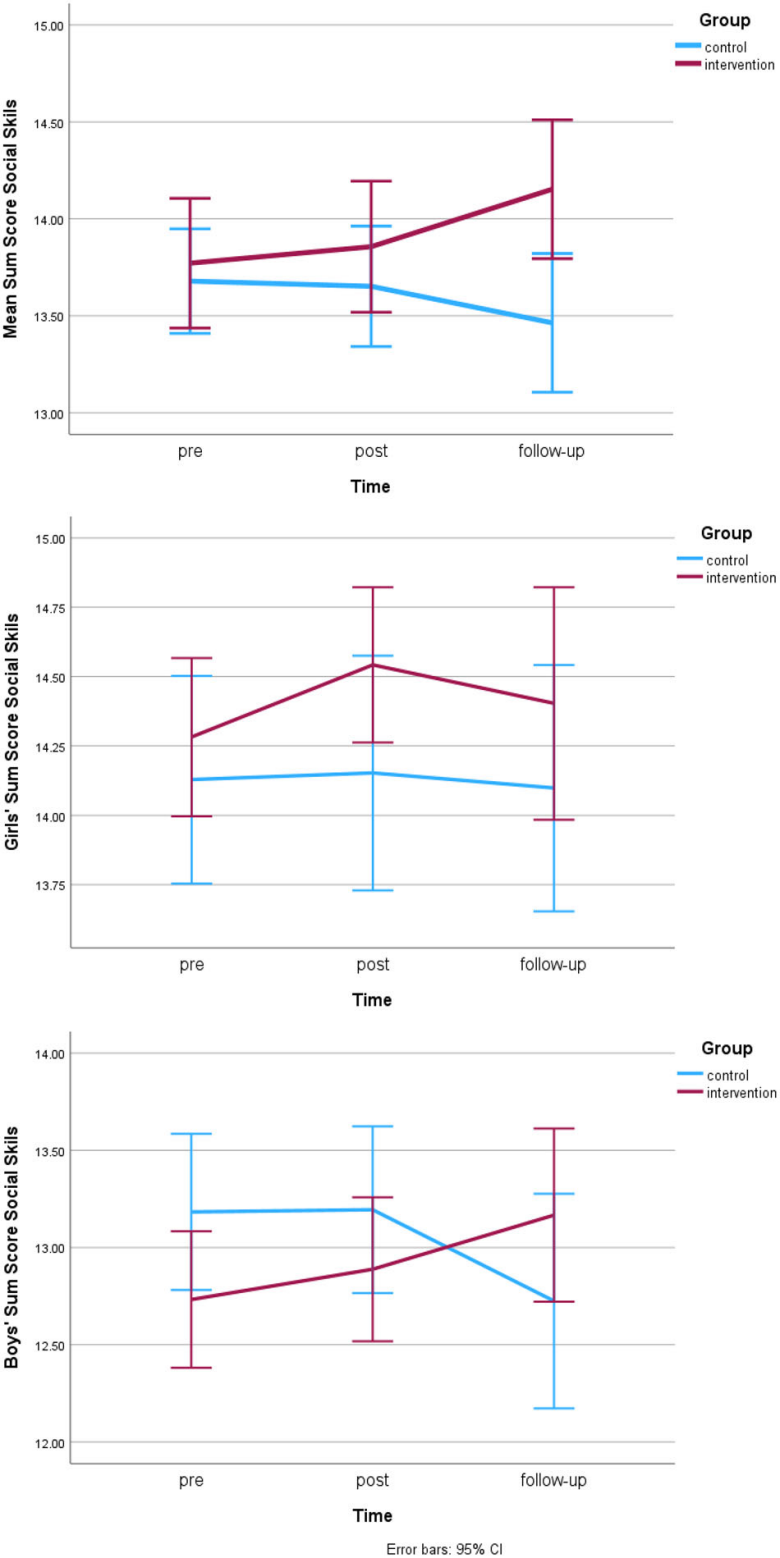
Changes in social-emotional skills across pre- post and follow-up

No pre-differences in social-emotional skills were found between intervention and control group. Over the intervention period (T1-T2), no significant main effects of time or group and no interaction effect were observed for social-emotional skills (see Table 4). However, during the follow-up period (T2-T3), a significant time x group interaction effect (*F*(1,365) = 6.83, *p* = 0.009, η²_p_ = 0.02, model fit BIC = 3479.64) was found. Similarly, over the whole study period (T1-T3), a significant time x group interaction effect (*F*(1,393) = 6.39, *p* = 0.012, η²_p_ = 0.02, model fit BIC = 3813.55) was observed. As evident from Fig. 1, the intervention group steadily increased their social-emotional skills levels with a particular increase from post to follow-up, while the control group slightly but steadily decreased (see also Table 3).

**Table 3:** Means and standard errors of all outcome variables for intervention and control group

Outcome variable	Time	Group	*M*	*SE*
Social Skills	*Pre*	control	13.68	0.14
		intervention	13.77	0.17
	*Post*	control	13.65	0.16
		intervention	13.86	0.17
	*Follow-up*	control	13.46	0.18
		intervention	14.15	0.18
Classroom Climate Student	*Pre*	control	26.51	0.32
		intervention	27.75	0.37
	*Post*	control	25.89	0.37
		intervention	26.53	0.40
	*Follow-up*	control	25.34	0.37
		intervention	26.34	0.44
Classroom Climate Teacher	*Pre*	control	31.86	0.26
		intervention	32.12	0.24
	*Post*	control	31.77	0.32
		intervention	32.08	0.28
	*Follow-up*	control	31.49	0.32
		intervention	32.56	0.26
Peer Connectedness	*Pre*	control	16.24	0.18
		intervention	15.92	0.22
	*Post*	control	16.36	0.21
		intervention	16.14	0.24
	*Follow-up*	control	16.09	0.21
		intervention	16.52	0.26
Happiness	*Pre*	control	16.13	0.19
		intervention	16.56	0.20
	*Post*	control	16.06	0.22
		intervention	16.29	0.23
	*Follow-up*	control	15.56	0.23
		intervention	16.50	0.25

Gender was added to the intervention effect model for the whole study period to analyze gender differences across the whole time period. This model yielded a significant main effect of gender (*F*(1,484) = 35.12, *p* < 0.001, η²_p_ = 0.07, model fit = 3686.49) and a significant three-way interaction between gender, group and time (*F*(1,390) = 4.15, *p* = 0.042, η²_p_ = 0.01). To further explore the main effect of gender, pre-test differences between the genders in social skills were analyzed. Indeed, significant pre-test differences were found (F(1,441) = 28.86, p = <0.001, η²_p_ = 0.06) with boys reporting lower pre-skills (*M* = 13.15, *SE* = 0.18) than girls (*M* = 14.29, *SE* = 0.18). To analyze intervention effects in detail for both genders, the data was split by gender and separate intervention effect analyses with time and group were run. While a significant time x group interaction effect on social-emotional skills was found for boys (*F*(1,185) = 9.02, *p* = 0.003, η²_p_ = 0.05, model fit BIC = 1882.36), this effect was not observed for girls. Descriptive statistics reveal that boys in the control group decreased in their social-emotional skills (pre *M* = 13.19, *SE* = 0.31, follow-up *M* = 12.66, *SE* = 0.28), while boys in the intervention group increased (pre *M* = 13.02, *SE* = 0.23, follow-up *M* = 13.59, *SE* = 0.35).

#### Classroom climate generated by students

**Fig. 2:**
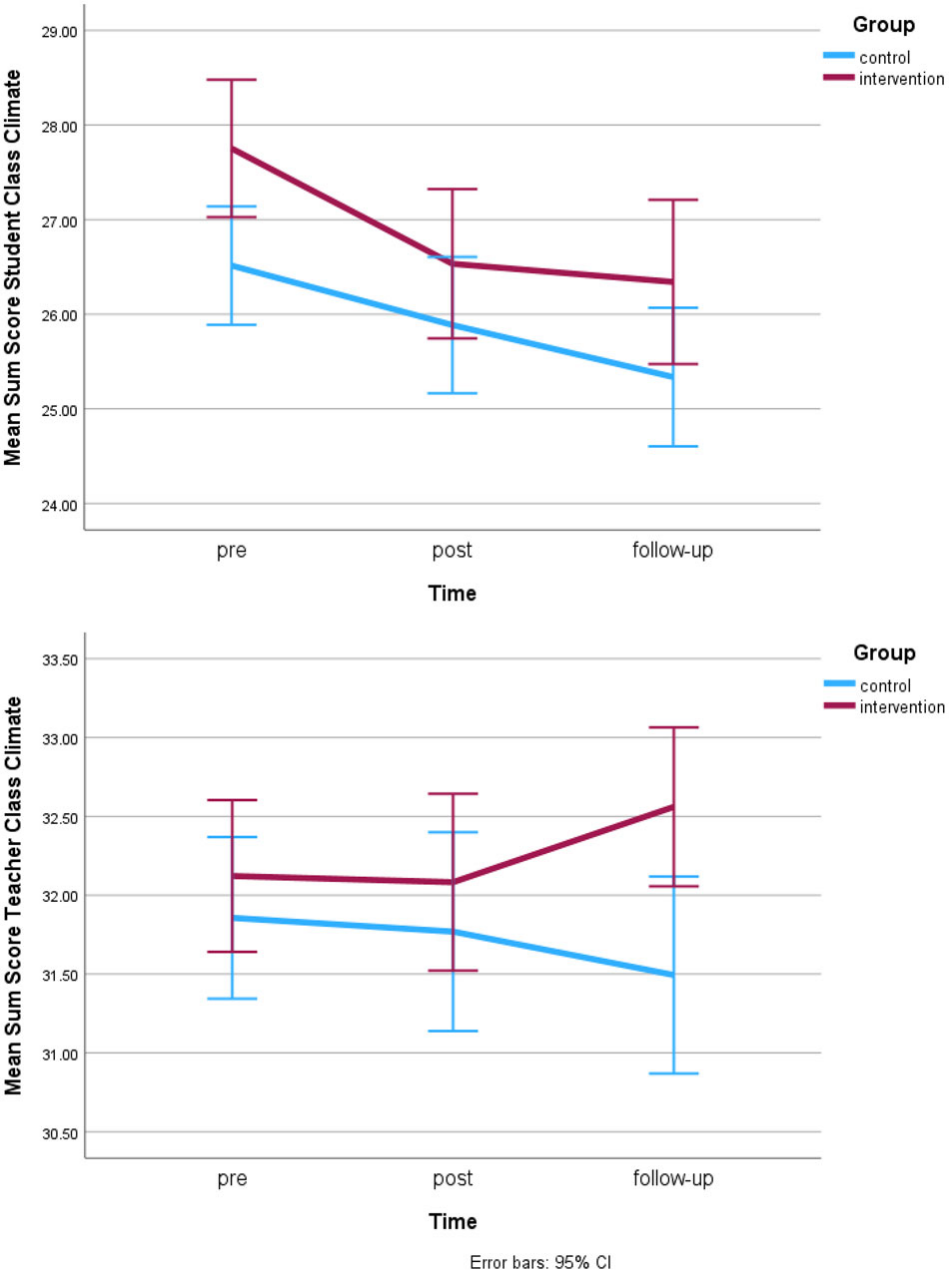
Changes in classroom climate generated by students and teachers across pre- post- and follow-up

At pre, no significant differences in student classroom climate between intervention and control group were observed. Over the intervention period (T1-T2), a significant effect of time (*F*(1,409) = 20.98, *p* < 0.001, η²_p_ = 0.05, model fit BIC = 5256.92) was observed, but no interaction effect (see Table 4). Similarly, over the whole study period (T1-T3), a significant main effect of time (*F*(2,412) = 20.29, *p* < 0.001, η²_p_ = 0.05, model fit BIC = 5252.68) was observed, but no interaction effect. Both, the intervention and control group, reported a decrease of class climate from pre to post. The intervention group was stable until follow-up, while the control reported further decreases (see Fig. 2), differences were however not statistically significant (see Table 3). No differential effects of gender were found.

**Table 4:** Results of main intervention effects analysis using linear mixed models

Outcome Variable	Multilevel Linear Model	T1-T2					T2-T3					T1-T3				
	Predictor Variable	df num	def denom	F	p	η²_p_	df num	def denom	F	p	η²_p_	df num	def denom	F	p	η²_p_
Social Skills	*main effect time*	1	405.83	0.024	0.877		1	365.58	0.084	0.772		1	393.364	0.553	0.458	
	*main effect group*	1	22.14	0.251	0.621		1	21.13	1.514	0.232		1	23.267	1.697	0.205	
	*interaction time x group*	1	405.83	0.429	0.512		1	365.58	6.829	0.009*	0.018	1	393.364	6.390	0.012*	0.015
Classroom Climate Student	*main effect time*	1	409.22	20.982	<0.001**	0.051	1	374.56	0.670	0.413		1	412.232	20.296	<0.001**	0.046
	*main effect group*	1	21.40	0.838	0.370		1	20.09	0.506	0.485		1	21.470	1.260	0.274	
	*interaction time x group*	1	409.22	2.132	0.145		1	374.56	0.483	0.487		1	412.232	0.309	0.579	
Classroom Climate Teacher	*main effect time*	1	418.51	0.051	0.822		1	380.90	0.005	0.945		1	398.859	0.038	0.845	
	*main effect group*	1	19.59	0.338	0.567		1	18.78	1.109	0.305		1	18.831	2.118	0.162	
	*interaction time x group*	1	418.51	0.008	0.928		1	380.90	3.392	0.066		1	398.859	3.158	0.076	
Peer Connectedness	*main effect time*	1	405.48	0.181	0.670		1	367.57	0.004	0.948		1	406.941	0.889	0.346	
	*main effect group*	1	21.19	0.612	0.442		1	21.07	0.000	0.984		1	22.278	0.000	0.985	
	*interaction time x group*	1	405.48	0.122	0.726		1	367.57	3.573	0.059		1	406.941	4.576	0.033*	0.011
Happiness	*main effect time*	1	400.23	2.569	0.109		1	371.38	0.165	0.684		1	393.365	6.242	0.013*	0.016
	*main effect group*	1	20.14	0.892	0.356		1	20.13	1.174	0.291		1	20.412	3.109	0.093	
	*interaction time x group*	1	400.23	0.740	0.390		1	371.38	7.400	0.007*	0.019	1	393.365	3.124	0.078	

*p* values in bold indicate statistical significance

*Indicates statistical significance at <0.05

**Indicates statistical significance at <0.001

#### Classroom climate generated by teachers

No significant pre-test differences between intervention and control group were found for teacher generated classroom climate. No significant main effects or interaction effects were found for intervention period (T1-T2), nor follow-up period (T2-T3), nor whole study period (T1-T3). While not reaching statistical significance, a trend for a time x group interaction (*F*(1,380) = 3.39, *p* = 0.066, η²_p_ = 0.01, model fit BIC = 4523.82) was observed over the follow-up period (see Table 4). As evident from Fig. 2, both groups were relatively stable between pre and post, but while the intervention group reported a slight increase, the control group reported a slight decrease at follow-up (see also Table 3). No differential effects of gender on perceived teacher classroom climate were found.

Hypothesis 1, which predicted direct intervention effects (T1-T2) on social-emotional skills, and student and teacher generated class climate, was therefore not supported. Although the significant time x group interaction effects for social-emotional skills indicate effects of the intervention, these effects became evident only when including the follow-up period (T1-T3/T2-T3). With no significant interaction effects for either class climate measure, no intervention effects were observed during the study period (T1-T2).

#### Peer connectedness

**Fig. 3:**
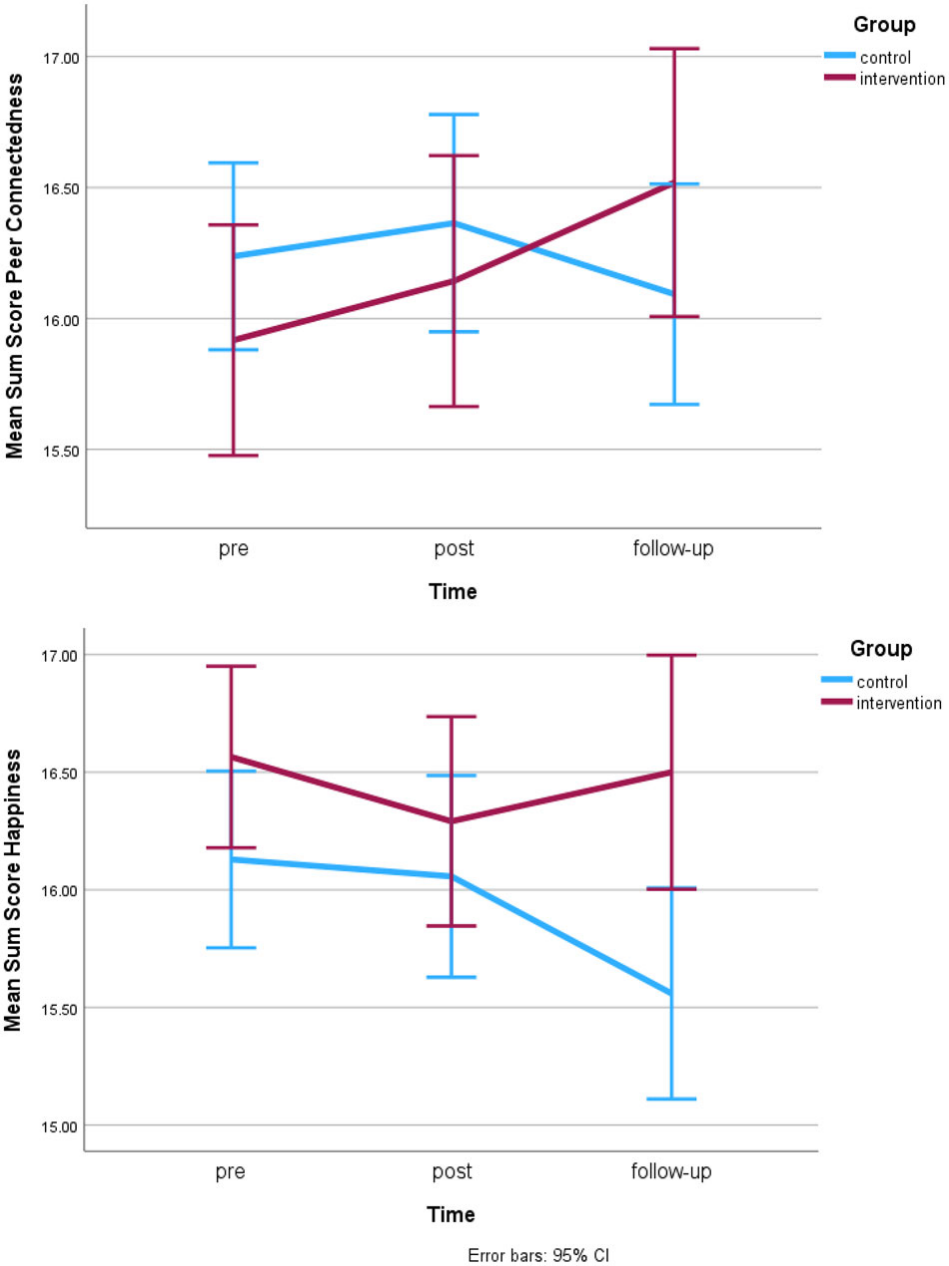
Changes in peer connectedness and happiness across pre- post- and follow-up

No pre-differences between intervention and control group were found for peer connectedness. No significant main effects of time or group and no significant interaction effects were found for the intervention period (T1-T2, see Table 4) or the follow-up period (T2-T3). While not reaching statistical significance, a trend for a group x time interaction effect was observed during the follow-up period (*F*(1,367) = 3.57, *p* = 0.060, η²_p_ = 0.01, model fit BIC = 4021.71). Over the whole study period (T1-T3) a significant interaction effect between group and time was observed (*F*(1,406) = 4.58, *p* = 0.033, η²_p_ = 0.01, model fit BIC = 4355,12). While both groups slightly increased in their levels of connectedness between pre and post, the intervention group reported further increases until follow-up, while the control group reported a decrease until follow-up (see Fig. 3 and Table 3). When adding gender to the T1-T3 effect model, a significant main effect of gender on connectedness was observed (*F*(1,485) = 9.11, *p* = 0.003, η²_p_ = 0.02, model fit BIC = 4174.1) with girls reporting higher connectedness than boys (girls overall *M* = 16.58, *SE* = 0.24, boys overall *M* = 15.79, *SE* = 0.24). However, no interaction between gender and intervention group or time was observed.

#### Happiness

No pre-differences between intervention and control group in happiness levels were observed. Over the intervention period (T1-T2), no significant effects were observed (see Table 4). Over the follow-up period (T2-T3), a significant time x group interaction effect was found (*F*(1,371) = 7.40, *p* = 0.007, η²_p_ = 0.02, model fit BIC = 3961.35). Over the whole study period (T1-T3), a significant main effect of time (*F*(1,393) = 6.24, *p* = 0.013, η²_p_ = 0.01, model fit BIC = 4339.93) was observed. While not reaching statistical significance, there was a trend towards a significant time x group interaction effect (*F*(1,393) = 3.12, *p* = 0.078) and a slight trend towards a main effect of group (*F*(1,20) = 3.11, *p* = 0.093). While both groups showed a slight decline in happiness from pre to post, the intervention group increased again until follow-up, while the control group further decreased (see Fig. 3 and Table 3). Including gender in the T1-T3 intervention effect model, yielded a main effect of gender (*F*(1,477) = 10.06, *p* = 0.002, η²_p_ = 0.02, model fit BIC = 4157.47), with girls reporting higher happiness levels than boys (girls overall *M* = 16.55, *SE* = 0.24, boys overall *M* = 15.71, *SE* = 0.24). However, no interaction between gender and intervention group or time was observed.

Hypothesis 2, which predicted significant intervention effects on happiness and connectedness after the intervention and follow-up, was partly supported. Again, no significant intervention effects were observed over the study period (T1-T2), however significant effects on happiness at follow-up were observed.

### The Impact of Implementation Fidelity on Intervention Effects

#### Effectiveness analysis with all implementation progress groups

To assess effectiveness of the intervention under different implementation progress conditions, an exploratory analysis with all implementation progress groups (full program, partly finished program, few sessions, drop-out and control group) was conducted. For the presented analysis, mixed linear models with group (intervention/control) and three levels of time (T1-T2-T3) were conducted, as this analysis was more exploratory compared to the main analysis, which tested specific predictions by considering intervention period and follow-up period separately.

Pre-test differences in some of the outcome variables indicated that the implementation groups already differed before the start of the intervention. For happiness, significant pre-differences (*F*(4, 28) = 3.02, *p* = 0.034, η²_p_ = 0.29, model fit BIC = 3447.74) were found. Post-hoc tests indicated that the drop-out group (*M* = 14.66, *SE* = 0.61) reported significantly lower happiness levels than the full program group (*M* = 16.56, *SE* = 0.34, *p* = 0.011, 95% C.I. = [0.46, 3.31]) and the control group (*M* = 16.05, *SE* = 0.26*, p* = 0.044, 95% C.I. = [−2.74, −0.04]). Similarly, classrooms that completed few sessions (*M* = 14.50, *SE* = 0.70) reported significantly lower happiness than full program classrooms (*p* = 0.014, 95% C.I. = [−3.64, −0.45]) and control classrooms (p = 0.048, 95% C.I. = [−3.07, −0.02]). For social-emotional skills, a strong trend (*F*(4, 30) = 2.63, *p* = 0.053, η²_p_ = 0.25, model fit BIC = 3032.24) indicated pre-test differences and post-hoc tests indicated that the drop out group (*M* = 12.24, *SE* = 0.46) reported significantly lower social-emotional skills than the full program (*M* = 13.78, *SE* = 0.25, *p* = 0.006, 95% C.I. = [−2.60, −0.48]) and the control group (*M* = 13.68, *SE* = 0.19*, p* = 0.006, 95% C.I. = [−2.45, −0.44]). For student and teacher classroom climate and connectedness, no pre-test differences were found. For variables with pre-test differences between groups (i.e., happiness and social-emotional skills), the drop-out (and few session) group was excluded from further analyses to allow the analysis to detect potential interaction effects generated by the intervention, unbiased by pre-test differences between groups.

**Fig. 4:**
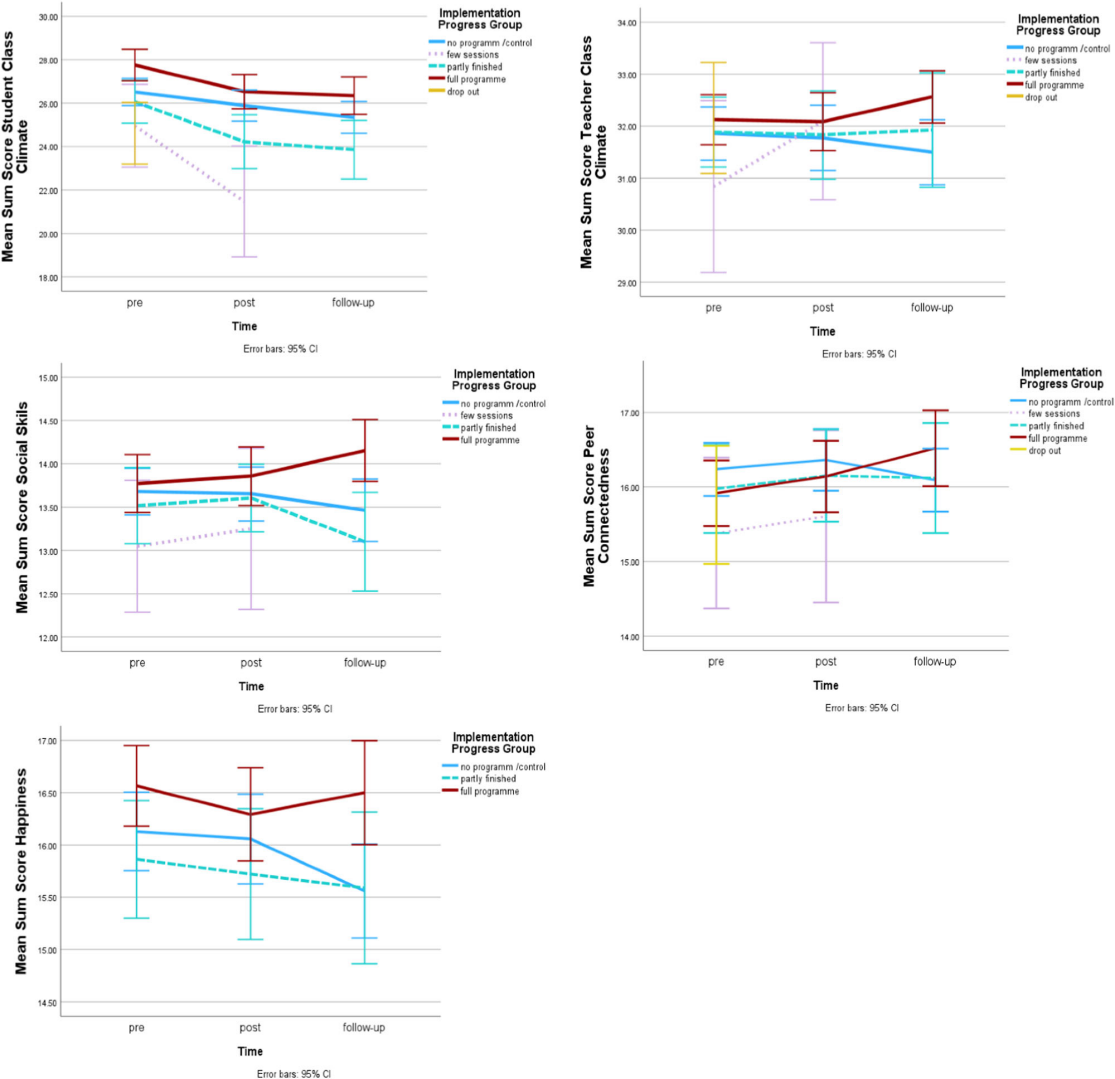
Changes of implementation fidelity groups on all outcome variables across pre- post- and follow-up

For social-emotional skills, a significant time x group interaction was found (*F*(5, 634) = 2.58, *p* = 0.025, η²_p_ = 0.02, model fit BIC = 7048.88). Post-hoc tests were not significant, indicating that the groups did not differ significantly at any specific time point. To further explore whether the significant *interaction* was driven by full program and control group (as found in the main analysis), or also present between full program and partly finished groups, a specific model with the full program group and partly finished group was run, which revealed a significant time x group interaction (*F*(2,266) = 5.59, *p* = 0.004, η²_p_ = 0.04, model fit BIC = 3548.13), with descriptive statistics indicating that the full program reported consistent increases (pre *M* = 13.75, *SE* = 0.27, post *M* = 13.79, *SE* = 0.27, follow-up *M* = 14.09, *SE* = 0.28) while the partly finished group reported an increase from pre to post, but a decrease in the follow-up period (pre *M* = 13.51, *SE* = 0.33, post *M* = 13.52, *SE* = 0.32, follow-up *M* = 13.06, *SE* = 0.35), similar to the control group’s trajectory reported in the main intervention effect analysis (see Fig. 4).

For student classroom climate, a significant time x implementation group interaction was found (*F*(5,603) = 2.72, *p* = 0.019, η²_p_ = 0.02, model fit BIC = 10230.59), as well as a main effect of time (*F*(2,548) = 31.09, *p* < 0.001, η²_p_ = 0.10) and – although not reaching statistical significance – a trend for a main effect of group (*F*(4,31) = 2.58, *p* = 0.056, η²_p_ = 0.25). All groups reported declines in their perceived classroom climate (see Fig. 4), but post-hoc tests indicated significant differences between the full program group and the few sessions group (*p* = 0.038, 95% C.I. = [0.20,6.95]) and a trend towards significant differences between the full program and the partly finished group (*p* = 0.097, 95% C.I. = [−0.37, 4.20]). Both, the few sessions group and the partly finished group reported lower pre-test climate (*M* = 24.98, *SE* = 1.50, and *M* = 26.32, *SE* = 0.88 respectively) and decreased further at post (*M* = 21.28, *SE* = 1.60, and *M* = 24.39, *SE* = 0.91 respectively) and follow-up (data available for partly finished group only *M* = 23.64, *SE* = 0.93), compared to the full program group (pre *M* = 27.69, *SE* = 0.74, post *M* = 26.21, *SE* = 0.76, follow-up *M* = 26.20, *SE* = 0.77).

For happiness, the implementation effect model yielded a main effect of time (*F*(2,503) = 3.31, *p* = 0.037, η²_p_ = 0.01, model fit BIC = 7683.59). While not reaching statistical significance, a trend for an interaction effect between time and implementation group (*F*(4,505) = 2.07, *p* = 0.084, η²_p_ = 0.02) was found. From pre to post, full program (pre *M* = 16.55, *SE* = 0.35, post *M* = 16.19, *SE* = 0.36), partly finished (*M* = 15.85, *SE* = 0.43, post *M* = 15.68, *SE* = 0.44) and control group (pre *M* = 16.03, *SE* = 0.27, post *M* = 15.88, *SE* = 0.28), all reported declines in their happiness. At follow-up, the control group (*M* = 15.41, *SE* = 0.29) as well as the partly finished group (*M* = 15.49, *SE* = 0.47) reported further declines, while the fully finished group (*M* = 16.46, *SE* = 0.38) reported increases (see Fig. 4). For peer connectedness and teacher classroom climate, no significant effects were observed.

#### Teacher motivation and class climate

Additionally, it was explored whether teachers, who reported higher perceived relevance of intervention contents, would be perceived as more engaging concerning the class climate by their students. Spearman correlation coefficient was computed to assess the relationship between teacher reports of their perceived relevance of contents and student reports of their perceived teacher generated class climate after the intervention period (T2). The relationship was negligible and not significant (r = −0.06, p = 0.83). For all correlations between fidelity measures and student perceived teacher climate see Appendix 1.

To explore whether students’ perceived teacher class climate predicted student class climate, a linear mixed model predicting the change in student class climate after the intervention period (T2-T3) by the change in teacher generated class climate during the intervention period (T1-T2) was run. As this analysis aimed to explore implementation effects, it included intervention classrooms only. Student class climate was not significantly predicted by teacher generated class climate, but a trend for significance was observed (F(1,224) = 3.65, p = 0.057, η²_p_ = 0.02, model fit BIC = 1340.60).

### Exploratory Analysis of Low Pre Skills and Connectedness

During the analysis, it became clear that pre-values of social-emotional skills and peer connectedness were skewed towards the top values. The social-emotional skills scale included 4 questions with a 4 point Likert-scale (1-never, 2-seldom, 3-sometimes, 4-regularly). At pre-test, 28.3% of children reported the maximum score of 16. The peer connectedness scale included 4 questions with a 5 point Likert-scale (1 and 2 - low connectedness, 3 -medium connectedness, 4 and 5 -high connectedness). At pre, one third of participants achieved a sum score of 18 or above (out of 20). This made overall increases less likely and more difficult to detect, thereby potentially causing an underestimation of the actual intervention effects (Chyung et al., 2020). Thus, an exploratory analysis was conducted to test whether children reporting lower skills and connectedness at pre, profited differently from the intervention. As about one third of children reported very high sum scores for both measures, the 33% percentile was used as reference value to create an equal distribution of children on the high and low end of the spectrum. For social-emotional skills, a cut-off point of 12 was chosen based on the 33% percentile and children scoring 12 or below at pre (total of 27.3%) were grouped as reporting low social-emotional skills. Within this group of children reporting low social-emotional skills at pre, 48 children were in the intervention group and 79 children in the control group. Within the group of children reporting high social-emotional skills at pre, 140 were in the intervention group and 198 were in the control group. For connectedness, a cut-off point of 15 was chosen based on the 33% percentile and children scoring 15 or below at pre (total of 36.6%) were grouped as reporting low peer connectedness. Within this group of children reporting low peer connectedness at pre, 73 children were in the intervention group and 97 children in the control group. Within the group of children reporting high peer connectedness at pre, 120 were in the intervention group and 181 were in the control group.

Children reporting lower social-emotional skills at pre, also reported significantly lower happiness levels (*F*(1,666) = 64.29, *p* < 0.001, η²_p_ = 0.09, model fit BIC = 3356.32), lower peer connectedness (*F*(1,665) = 114.40, *p* < 0.001, η²_p_ = 0.15, model fit BIC = 3290.77), lower perceived classroom climate (*F*(1,664) = 82.17, *p* < 0.001, η²_p_ = 0.11, model fit BIC = 4030.27) and lower teacher generated class climate (*F*(1,663) = 65.67, *p* < 0.001, η²_p_ = 0.09, model fit BIC = 3699.01) compared to children reporting higher social-emotional skills. Similarly, children reporting lower peer connectedness at pre, also reported significantly lower happiness levels (*F*(1,669) = 74.17, *p* < 0.001, η²_p_ = 0.10, model fit BIC = 3387.88), lower social-emotional skills (*F*(1,663) = 99.39, *p* < 0.001, η²_p_ = 0.13, model fit BIC = 2951.54), lower perceived classroom climate (*F*(1,661) = 111.65, *p* < 0.001, η²_p_ = 0.14, model fit BIC = 4027.63) and even lower teacher generated class climate (*F*(1,668) = 37.40, *p* < 0.001, η²_p_ = 0.05, model fit BIC = 3734.88) compared to children reporting high peer connectedness.

To test whether children reporting lower social-emotional skills at pre profited differently from the intervention, the data used in the main intervention effect analysis (“full program” and control groups) was split between high and low pre-social-emotional skills and a model predicting skills change scores by the intervention/control group was tested separately for the high and low pre skills group. As the main intervention effect analysis found intervention effects to become apparent in the follow-up period or whole study period, the following analysis focused on these periods only (T1-T3/T2-T3).

Over the whole study period (T1-T3), the intervention did not predict social-emotional skills change scores for either pre skills group. Over the follow-up period (T2-T3), however, the intervention did predict social-emotional skills change scores (*F*(1,241) = 4.66, *p* = 0.032, η²_p_ = 0.02, model fit BIC = 993.56) for the high pre skills group. In the control group, children, who had reported high social-emotional skills at pre, reported a decrease of social-emotional skills (T2-T3 change score *M* = −0.32, *SE* = 0.16), whereas those in the intervention group reported an increase (T2-T3 change score *M* = 0.19, *SE* = 0.17).

To test whether children reporting lower connectedness at pre profited differently from the intervention, an equivalent approach was taken with connectedness scores as for social-emotional skills reported above. The intervention group did not significantly predict connectedness change scores in the study period (T1-T3) or follow-up period (T2-T3) for either group. Thus, no differential intervention effects seem to exist in relation to reported pre connectedness scores.

### Testing the Intervention Effect Mechanisms

The relationships between target variables in the intervention group, it was hypothesized that changes in social-emotional skills during the intervention period (T1-T2) would predict changes in classroom climate and peer connectedness in the follow-up period (T2-T3). However, over the intervention period (T1-T2), negligible changes in social-emotional skills were observed. As the aim of this analysis was to understand the relationship between the changes in target variables affected by the intervention (to specify an intervention effect model), the analysis was modified to focus on time periods which were found to be subject to changes in the target variables (as reported in the main intervention effect analyses). Thus, it was assessed whether changes in social-emotional skills over the whole study period (T1-T3) correlated with changes in peer connectedness over the whole study period (T1-T3). This analysis does not provide implications concerning a chain of effects over time as the initially planned analysis would have. However, as social skills were not immediately impacted over the intervention period, the adapted analysis better reflects the relationship between simultaneously affected target variables. Classroom climate had to be dropped from this analysis, because it was not found to be affected by the intervention. Similarly, as significant effects on connectedness were observed for the whole study period (T1-T3), but not for the follow-up period (T2-T3) as originally hypothesized, it was assessed whether observed changes in connectedness over the whole study period (T1-T3) correlated with the observed changes in happiness in the follow-up period (T2-T3). This analysis aimed to assess mechanisms driving intervention effects and thus included the “full program” group only (n = 195) as it was assumed that classes that had not completed the intervention would not display full intervention effects. Due to ceiling effects and reported different intervention effects for low and high pre social-emotional skills groups, additional analysis took pre social-emotional skills and pre connectedness values into account.

Across all “full program” children, and over the study period (T1-T3), changes in social-emotional skills were found to correlate significantly with changes in connectedness (*F*(1,150) = 13.42, *p* < 0.001, *r* = 0.28, model fit BIC = 793.03). When analyzing the groups of low and high pre skills separately, this correlation was found for both, children with low pre-social-emotional skills (*F*(1,31.94) = 19.88, *p* < 0.001, *r* = 0.75, model fit BIC = 594.79) and high pre-social-emotional skills (*F*(1,108) = 5.29, *p* = 0.023, *r* = 0.22, model fit BIC = 199.69). The direction of effect was positive for both groups, indicating that increases in social-emotional skills correlate with increases in connectedness.

Next, effects of connectedness changes on happiness were evaluated. Across intervention (“full program”) children, changes in connectedness over the whole study period (T1-T3) were not found to significantly predict follow-up changes in happiness (T2-T3). A very slight trend towards significance was observed (*F*(1,145) = 2.90, *p* = 0.091, *r* = 0.14, model fit BIC = 661.44). When analyzing the groups of low and high pre connectedness separately, though, a significant effect of connectedness changes on happiness changes (*F*(1,95) = 4.27, *p* = 0.042, *r* = 21, model fit BIC = 437.54) was observed in the group of children with high pre connectedness levels. Children reporting high pre connectedness were more likely to report an overall decrease of connectedness (*M* = −0.45, *SE* = 0.29) as well as happiness (*M* = −0.20, *SE* = 0.26) and the direction of the effect was positive. Thus, the results suggest that a decrease in connectedness led to a decrease in happiness. No effects were found for the group of children reporting low pre connectedness levels.

Hypothesis 3 and 4, predicting that increases in social-emotional skills would predict increases in class climate and connectedness (H3), and that connectedness would predict happiness (H4) were partly supported. Social-emotional skills increases were found to correlate significantly with connectedness increases over the whole study period, with a specifically high correlation for children reporting low pre skills. Connectedness changes were found to significantly predict happiness changes, but only for children reporting high pre connectedness. Thus decreases in connectedness seem to significantly predict decreases in happiness.

## Discussion

To address a gap in the evidence base regarding the relationship between target variables of social-emotional learning programs after the school transition, a novel social-emotional learning program for 9 to 12-year- olds was developed. Apart from evaluating its effects on social-emotional skills, classroom climate, peer connectedness and happiness, an intervention effect model was developed to examine the relationship between its target variables. The present study’s results of significant intervention effects on social-emotional skills and peer connectedness over the whole study period (T1-T3) as well as on happiness over the follow-up period (T2-T3) support the notion that universal, school-based social-emotional learning programs have the potential to facilitate peer relationships (Suldo et al., 2013) and happiness in the classroom (Heinsch et al., 2020). However, the intervention effect model, postulating that social-emotional skills increases would predict increases in peer connectedness, while such increases in peer connectedness would predict increases in happiness was only partly supported.

### Main Intervention Effect Analysis

While it was hypothesized that the intervention would directly impact social-emotional skills over the intervention period (T1-T2), effects on social-emotional skills only became evident over the whole study period. Similarly, the present study’s effects on peer connectedness became evident over the whole study period (T1-T3) and effects on happiness become evident in the follow-up period (T2-T3) only. This is, however, not uncommon. The authors of other intervention studies, which found effects to become evident at follow-up only, suggested it might simply take longer for competencies to unravel or effects of high quality implementation might lead to better maintenance over time (Caron et al., 2020). This might be particularly true for peer relationship programs. It has been reported that 12% of students did not have any friends several months after the school transition (Lessard & Juvonen, 2018). Thus, to understand the long-term effects of social-emotional skills programs on peer relationships, longitudinal studies are essential (Gutman & Schoon, 2015). The present study’s intervention period of six months was able to capture some of the complex developmental trajectories. However, it has been argued that even a 2-year follow-up might not be sufficient to capture all effects (Mackenzie & Williams, 2018), due to this impactful developmental period and non-linear growth trajectories of target skills (Ross et al., 2019).

The findings of this study also point towards common negative trajectories after the school transition. An exploratory analysis of children reporting high and low pre-social-emotional skills showed that the intervention particularly helped to prevent decreases in social-emotional skills for children who reported high skills at pre. In the control group, these children tended to report a decline in skills. Similarly, significant effects of time on happiness suggested a general decline in happiness over the study period, although significant intervention effects over the follow-up period showed that the intervention was able to counterbalance this common trajectory to some degree. These trajectories are particularly interesting, as classroom climate generated by students was found to significantly decline for both the intervention and control group, contrary to this study’s hypothesis. Other studies have shown that children’s wellbeing, happiness and perceived school climate is highest in primary school and decreases after the transition (Coelho et al., 2020; Eder, 2007; Tobia et al., 2019). Many school climate factors, such as school safety, school liking and teacher-student relationships seem to decline with the transition (Coelho et al., 2020). In the present study, children were asked to complete pre-measures just after the transition in September, thus they might have answered questionnaires with their primary school classroom in mind and expected to replicate the same class climate in their secondary school. After settling into their secondary classroom they might have answered the post and follow-up questionnaires more realistically matching their secondary classroom – thus this study’s finding of a decline of class climate and happiness might just reflect differences in primary and secondary school classroom climate.

Similarly, a reported decline in social skills in the control group might reflect children’s process of adjusting their self-perception from the previous primary school context to the new secondary school context. With secondary schools focusing more on academic factors compared to students’ emotional needs (van Rens et al., 2018), children have previously reported changes in their relationships with peers and teachers and that it was harder to share problems in secondary school (Bagnall et al., 2020). Thus, the move to a new classroom might also impact children’s self-perception of their social-emotional skills due to early adolescents’ heightened focus on group norms (Hazen et al., 2008) and classroom norms impacting social behavior and acceptance (Chang, 2004). In the intervention group, however, children seemed to retain their positive perspective of their own skills, either due to an actual increase in skills through the intervention or due to more positive classroom interactions which led to more individual prosocial behavior (Busching & Krahé, 2020). Similarly, significant intervention effects on happiness in the follow-up period suggest that the intervention was able to counterbalance negative transition effects.

Why the intervention seemed to be able to retain children’s happiness and perception of their social-emotional skills, while not increasing the classroom climate, was not immediately clear. In the context of SEL programs’ outcomes, it has been suggested that awareness-competencies are preconditional for changes in action-focused competencies (van de Sande et al., 2019), thus changes in children’s self-perceptions (regarding their wellbeing and skills) might change before they are able to implement specific prosocial actions in the classroom. As the classroom climate measure consisted of items referring to other students’ behavior in class, it might just take longer for effects to manifest on a group level (Caron et al., 2020). Additionally, it should be noted that children’s perceptions of the dynamics in their classroom are likely also dependent on structural school aspects. School climate has been found to be determined by institutional as well as safety aspects in addition to the community and academic aspects (Wang & Degol, 2016). Thus, a variety of studies have suggested systems-approaches to addressing school and classroom climate (Littlecott et al., 2019; Stirling & Emery, 2016), with social-emotional competency trainings being only one part of multiple strategies (Voight, 2015).

Additionally, gender differences became evident in social-emotional skills and peer connectedness. The intervention significantly improved social-emotional skills in boys, while no significant effects were found for girls. This is not surprising as normative growth trajectories of general SEL skills have been found to differ between genders with boys reporting a decrease in their social-emotional skills around the age of eleven before they increase again up until late adolescence (Ross et al., 2019). In line with this, the present study found boys in the control group to slightly decline, thus a school-based intervention seems to have the potential to reverse negative trajectories of social-emotional skills in boys. For girls, however, increases beyond developmentally expected positive trajectories seem less feasible. Additionally, gender differences were found for connectedness with girls reporting higher overall connectedness than boys. General growth trajectories of relationship quality support this gender difference, with girls increasing their relationship quality from early to mid-adolescence and boys decreasing (Ross et al., 2019).

Generally, reported effect sizes are small. However, effects in psychological research are rarely large (Funder & Ozer, 2019) and it has thus been suggested to compare intervention effects to similar intervention studies (Hill et al., 2008). A meta-analysis of universal school-based interventions on wellbeing found effects to be generally small (Tejada-Gallardo et al., 2020). This might have to do with the preventive nature of universal interventions, which may have little effect on each individual as only few participants will be at-risk, although there is likely a significant cumulative benefit on the community level (Greenberg et al., 2017). On the contrary, targeted interventions for at-risk populations might yield higher effects, but are less likely to have a profound impact on the community (Greenberg et al., 2017). Moreover, for school-based interventions, a trend towards smaller effects of high quality studies has been reported (Mackenzie & Williams, 2018). Additionally, independent narrow measures, such as the ones used in this study, have been found to yield smaller effect sizes compared to broad or researcher-developed measures (Wolf & Harbatkin, 2023). It has been suggested that studies with large samples and small reported effect sizes are likely to reflect real-world circumstances authentically and that small effects can have profound real-world consequences when considering individual effects on a population level or single-occasion effects in the long-run (Funder & Ozer, 2019).

### Insights Concerning the Intervention Effect Mechanisms

It was hypothesized that, in the intervention group, increases in social-emotional skills would predict increases in connectedness and class climate (H3), which would in turn predict happiness (H4). This study’s findings partly supported these hypotheses. Due to a lack of immediate effects over the study period (T1-T2), the analysis concerning the relationships between target variables had to be modified. Instead of exploring relationships between intervention period changes and follow-up changes, it now focused on the changes found in the main intervention effect analysis and thus explored correlations of changes across the full study period to explore simultaneous changes in target variables. Increases in social-emotional skills were found to correlate significantly with increases in connectedness with specifically high correlations for children who reported low pre-skills. While this finding might be partly explained by the ceiling effects for pre-social-emotional skills, it also seems plausible that specifically children that have skills deficits and subsequently improve their prosocial behaviors gain new friendships (Bowker et al., 2010; Laugeson et al., 2014), and increase their connectedness.

Regarding the impact of changes in connectedness on changes in happiness (H4), a significant effect was found for children with high pre-connectedness only. As connectedness changes in children with high pre-connectedness are more likely to be negative, these results suggest that a decline in connectedness (i.e., loosing friends or decreased relationship quality) might have a greater impact on children’s happiness than gaining connectedness. The fear of being alone in the new class has been identified as a prevalent fear at the school transition (Stiehl et al., 2023) and indeed, the presence of a best friend at a negative experience has been found to alleviate some of the negativity, emphasizing the importance of close friends in the classroom (Adams et al., 2011). This relation between the experience of negative events without the supporting presence of a friend might explain why specifically a decrease in connectedness seems to be related to a decrease in happiness in class.

### Implementation Fidelity

To account for implementation factors during the evaluation (Skivington et al., 2021) and to address the relationship between implementation factors and effectiveness results (de Leeuw et al., 2020), an exploratory analysis evaluated the effectiveness of the intervention in different stages of implementation, reflecting adherence (i.e., duration and frequency of implemented sessions) as the main delivery aspect (Carroll et al., 2007). For happiness and social-emotional skills, pre-test differences existed between those classes that ended up completing the program and those that dropped out of the study (and classes completing few sessions only in the case of happiness). In classes with pre-existing problems, more trouble-shooting and targeted efforts might be necessary, which take up extra time and thus prevent teachers from finishing the program or cause early drop-out. These baseline differences are likely to impact the respective variables’ trajectories. Indeed, trajectories of social-emotional skills, classroom climate and happiness differed between groups. Generally, classrooms completing fewer sessions reported less favorable outcomes, with even the group of classes partly finishing the program reporting significantly lower social-emotional skills than the fully finished group. However, as fidelity analysis (see Appendix 1) showed, teachers that partly finished the program spent on average more time on each program session and reported a higher content coverage compared to teachers finishing the program. This suggests these teachers might not have managed to finish all program sessions, as they spent more time on the first sessions. Researchers also had the impression in telephone check-ins that teachers who reported having spent extra time on delivering basic concepts to their students (e.g., emotional vocabulary) would eventually not manage to finish all sessions.

These findings suggest it was not poor implementation fidelity impacting student outcomes, but pre-existing student factors impacting implementation fidelity. Unfortunately, these effects are likely to spiral, when classes with pre-existing problems receive less support. Classes with high levels of disruptive student behaviors and high teacher stress have been found to particularly benefit from interventions (Tolan et al., 2020). Thus, exactly the classes reporting implementation issues should be the focus of researchers and intervention developers. Reasons for these differences are often structural and related to school characteristics and socio-economic inequalities. At the most basic level, schools with more funding are able to better support the school transition by organizing more events (Evangelou et al., 2008). Teachers often refer to time restrictions and inflexible curricula as barriers to incorporating social-emotional learning in their class (Martínez, 2016). Schools that facilitate regular training are likely to offer teachers more support and empower them to successfully implement an intervention program (Domitrovich et al., 2008), which is even more essential when there are pre-existing classroom problems.

### Teacher Motivation and Class Climate

The main intervention effect analysis did not find any significant intervention effects for teacher generated classroom climate, as reported by their students. This variable was included for an exploratory analysis of the effects of teachers’ perceived relevance on teacher generated class climate and, in turn, on student generated class climate. Teachers’ perceived relevance and teacher generated class climate were not found to correlate significantly, and student generated class climate was not predicted by teacher class climate. While some previous studies have found such effects (Hellmich et al., 2019; Wilson et al., 2022), the findings of the present study are in line with another study that did not find teacher attitudes to predict their classroom management behavior (Garrote et al., 2020). It has been argued, that classroom management behavior might reversely impact teachers’ attitudes and that teachers’ self-efficacy might be a stronger predictor of behaviors (Garrote et al., 2020), which is supported by findings suggesting a mediator role of intervention deliverers’ self-efficacy on student outcomes (Caron et al., 2020). This emphasizes the importance of implementation support strategies at a school and policy level (Domitrovich et al., 2008). The non-significant effect of teacher generated class climate (i.e., teachers’ encouraging behaviors) on student generated class climate is likely related to the reported overall decrease of classroom climate. Secondary school teachers’ best efforts to create a positive class climate might not be able to outbalance students’ perceived decrease of class climate from primary to secondary school (Coelho et al., 2020; Tobia et al., 2019).

### Implications for Intervention Development and Educational Practice

This study’s findings regarding the effect mechanisms of a social-emotional learning program suggest that relationships between social-emotional skills and connectedness and happiness do exist. While social-emotional learning programs are commonly used to facilitate peer relationships in clinical populations (Pollak et al., 2022), similar approaches have been suggested as an ideal universal prevention (Greenberg et al., 2017) to improve peer relationships at the time of school transition (Suldo et al., 2013). This study’s findings support this notion, which is particularly relevant as this study also highlighted common negative trajectories of skills, classroom climate and happiness after the transition. Thus, the implementation of adequate social-emotional support programs seems highly relevant after the school transition to support children’s peer relationships and wellbeing (Bagnall et al., 2020; Rice et al., 2015).

The hypothesized intervention effect mechanisms (i.e., effects of social-emotional skill changes on connectedness, and connectedness changes on happiness) only came into effect under certain conditions. Thus, educators and program developers should carefully consider which aims can be addressed by different program clusters (e.g., universal SEL) for different populations (Pollak et al., 2022). Social-emotional learning programs were found to have strongest effects on self- and social awareness (van de Sande et al., 2019), which therefore seem like an ideal primary target skill. The present study found that many children report high baseline social-emotional skills, thus not all children will benefit from such a universal, preventive approach. However, the present findings suggest that an increase in social-emotional skills, which is commonly achieved in SEL programs (Gutman & Schoon, 2015), and was found to be achieved in this evaluation study, is significantly correlated with increased in peer connectedness. A particularly strong correlation between skills and connectedness seems to exist for children reporting social-emotional skills increases from lower baseline levels. As argued in the context of interventions for neurodiverse children or peer-rejected children, intervention efforts aiming to facilitate social-emotional skills of children with respective deficits are an effective way of increasing peer relationships in the classroom by (a) promoting target children’s skills and (b) helping peers with higher social-emotional skills better understand their peer’s needs (Kasari et al., 2016; Mikami et al., 2005). Thus, an emphasis on inclusion of struggling children and an empathic understanding of each other’s problems is suggested if educators’ focus is on increasing connectedness in the classroom. Similarly, the finding that happiness seems to be particularly impacted when high pre-connectedness decreases, points towards the importance of an inclusive class community. Friendship quality has been found to buffer against negative impacts of victimization (Cuadros & Berger, 2016) and trajectories of more withdrawn and excluded children to increased depression levels have been found to be minimized by the presence of a friendship (Bukowski et al., 2010). Thus, no child should be completely left out to ensure each child’s happiness in the classroom. While the proposed benefits might seem very specific and relevant for few children, such preventive efforts can have profound effects on a school or community level (Greenberg et al., 2017).

Furthermore, this study’s findings regarding the relation between pre-existing classroom problems and implementation fidelity, highlight the need of targeted adaptations and increased support systems for interventions. While most evaluation studies pursue maximum implementation fidelity, adaptation and adjustments have even been suggested to lead to better overall implementation (Durlak & DuPre, 2008). Teachers indicated that programs are only sustainable if they are accepted by students, feasible and linked to professional development opportunities (Boardman et al., 2005). Thus, researchers developing and evaluating interventions should account for adaptability and flexibility in their implementation protocols. Instead of disregarding drop-out classes, continued implementation support should be provided, potentially accepting minimized adherence. Additionally, schools should consider shared decision-making processes and providing resources, administrative support and certified training to enhance implementation (Domitrovich et al., 2008). These implications equally relate to this study’s findings that teachers’ attitudes do not relate to their classroom behavior. Even teachers perceiving social-emotional learning as highly relevant might struggle to implement it in their class without appropriate support.

### Strengths and Limitations

In this study, the program was implemented by the class teachers themselves, which is considered a strength. On a practical level, teacher-implementation was necessary due to Covid-19 related access restrictions at schools and economic considerations. Practical considerations are, however, related to ecological validity and sustainability of an intervention. Teacher-led implementation has been found to be particularly cost-efficient (Domitrovich et al., 2017), more effective as compared to external deliverers (Durlak et al., 2011) and preferred by students (Mackenzie & Williams, 2018). While direct observation would have been the best measure of fidelity (Gresham et al., 2017), conducting video calls for each session in each classroom would have been logistically impossible. Thus, a sampling of fidelity video calls of some sessions would have been the only option. As it is unclear how sampling and scheduling of observation sessions would affect the implementation by teachers (Barnett et al., 2014), researchers decided against this option to increase consistency of implementation. Additionally, teacher self-report of implementation practice has been found to be reliable and accurate (Dart et al., 2020), with a generalization study indicating that 7 instances of self-report over a few weeks are needed to create reliable data (Gresham et al., 2017), which was the case for the “full program” group. A support system consisting of a detailed manual, teacher training sessions and repeated telephone check-ins was implemented to monitor the program delivery and allow for a replication of this implementation (Domitrovich et al., 2008). While it has been suggested that school-based prevention programs are generally not implemented with high quality outside of research projects (Domitrovich et al., 2008), teacher-lead implementation is likely to counterbalance such research-biases as the implementation during the study is as close to large-scale, unsupervised implementation as possible, making it ecologically valid.

However, this implementation strategy also led to some imbalances between groups. Due to unbalanced drop-out, the intervention group had more grammar school classes than the control group. Teachers, who dropped out of the study mostly referred to time and resource issues. However, school resources are typically linked to the socio-economic profile of schools, which seems to be associated with students’ resilience outcomes (Agasisti et al., 2018) and predict students’ academic achievement beyond individual socio-economic background (Perry & McConney, 2010). Thus, this unbalanced drop-out might have impacted the results. At the same time, this bias is likely to be linked to previously discussed structural inequalities leading to teachers of classes with more problems also having less resources and thus not being able to finish the program. Indeed, seven out of eight teachers, who managed to finish the program, were grammar school teachers.

Similarly, structural differences might have impacted the timing of the follow-up period, which might have biased follow-up results. As follow-up measures were completed in a set calendar time period (late January, early February 2022), the follow-up period was stretched for classes finishing the program early and thus having an early post assessment (and eventually comprising the “full program” group). The remaining classes (eventually comprising “partly finished” and “few sessions” group) were asked to complete post-test just before Christmas, thus creating a short follow-up period. For similar reasons, the “full program” group had a longer follow-up period than the control group. As developmental changes (both facilitated by intervention effects as well as initiated by the school transition and age-related development) become more apparent over longer time periods, reported superior effects of the full program over the partly finished group and over the control group in the follow-up period might be impacted by the time frame. While the authors realize this is an important confounding factor, the longer follow-up period in the full program group is regarded a side effect of this group’s pre-existing advantage. If implementation runs smoothly due to high pre-skills and more school resources, the intervention is quick to be completed and an early post-test follows.

Additionally, as for any school-based study, methodological problems, such as the difficulty of randomizing school classes, regulating the control group’s school practices and the choice of outcome measures might impact effects (Mackenzie & Williams, 2018). The context of Covid-19 further complicated matters, as all teachers reported increased workload, stress (Robinson et al., 2023) and mental health problems of students (Theberath et al., 2022) coming out of long lockdowns. This increased school-specific responsibilities (concerning Covid-19 testing) as well as special school projects to replace/substitute typical school trips at the start of the year, which were impacted by Covid-19 regulations. To make an intervention start in September possible under these conditions, schools were allowed to choose their preferred group assignment (intervention/control) and control classes could not be prohibited from addressing social-emotional needs. Independent narrow measures were chosen to decrease variation of the distribution of latent true effects (Wolf & Harbatkin, 2023), considering validation results for the age group and German language, if possible. Despite these considerations, ceiling effects were found for social-emotional skills and connectedness, thus potentially biasing results. Additionally, this study aimed to consider a variety of implementation aspects, however, major differences in adherence dominated the implementation analysis. As this study’s findings suggest that these differences are structural, future intervention efforts should compensate such differences by offering adaptations (Durlak & DuPre, 2008) and school-level support (Domitrovich et al., 2008) to increase implementation quality. By preparing for these implementation factors, suggested in-depth analyses of the impact of organizational factors at the school-level (Jindal-Snape et al., 2020) and a more detailed examination of time and economic resources for each implementation stage (Premachandra & Lewis, 2021) might be possible.

## Conclusion

To address mixed results concerning the effects of school-based social-emotional learning programs and understand their effect mechanisms, a novel social-emotional learning program and the relationship between its target variables was evaluated. It was expected that the program would facilitate an increase in social-emotional skills, which would predict increases in connectedness and class climate, which would predict happiness. This study found positive intervention effects on social-emotional skills, peer connectedness and happiness. Hypothesized relationships between target variables were partly supported with significant correlations between social-emotional skills and peer connectedness increases, particularly for children with lower baseline skills. However, changes in connectedness were only correlated with changes in happiness in the case of children reporting a decrease of previously high connectedness. The findings support the notion that school-based social-emotional-leaning programs are effective universal, preventive approaches and have the potential to support peer relationships in the context of the school transition. However, decreases in class climate and happiness after the school transition highlight the need to implement more support programs over the school transition period. Additionally, an implementation progress analysis suggested baseline differences between classes that could complete all sessions and classes that dropped out, suggesting more implementation support is needed to ensure all children can benefit from support programs.

### Supplementary information

Supplementary Information

## Footnotes

1 In the Austrian school context, there are two types of secondary schools. (Lower grade) grammar schools span year 1 to year 4 and cater to students transitioning to higher grammar school, aiming to complete a high school diploma qualifying them for university. Comprehensive schools span year 1 to year 5 and cater to all students, either transitioning to vocational trainings after year 5 or to higher grammar schools after year 4, aiming to complete a high school diploma qualifying them for university. Admission to grammar schools largely depends on students’ grades in year 4 of primary school. However, the percentage of students enrolling in each school type varies by location, as comprehensive schools are more common in rural areas, while grammar schools are mostly located in urban areas. Therefore, students in rural areas commonly attend local comprehensive schools and later transition to higher grammar schools.

2 This analysis aimed to assess potential interaction effects between time and group across specific time points (T1-T2 / T2-T3). Fitting two separate models seemed more appropriate than having one general model with all time points, as a significant interaction effect across all time points would not differentiate where the groups changed over time (i.e., whether the groups changed differently between T1-T2 or T2-T3).
