# Supplementary material for: Promoting Peer Connectedness Through Social-Emotional Learning: Evaluating the Intervention Effect Mechanisms and Implementation Factors of a Social-Emotional Learning Programme for 9 to 12-Year-Olds
Source: J Youth Adolesc. 2023 Oct 5;53(1):89–116. doi: 10.1007/s10964-023-01871-x (PMC10761397; doi:10.1007/s10964-023-01871-x)
Supplement: Supplementary file 1 — Supplementary Information [file 10964_2023_1871_MOESM1_ESM.docx]

**Appendix A**

**Promoting peer connectedness through social-emotional learning: Evaluating the intervention effect mechanisms and implementation factors of a social-emotional learning programme for 9 to 12-year-olds**

[author names withheld for blind review]

**Detailed Results of Fidelity Measures**

A fidelity strategy was developed based on Caroll et al. (2007). To collect data about adherence to intervention protocol, teachers were asked to report about frequency and duration of their programme implementation and were additionally asked “Please indicate how detailed you covered the suggested content for this session?” (to be rated on a scale from 1-left out completely, 2-discussed in less detail, 3- completed, 4- discussed in more detail, 5- discussed very thoroughly). In open questions teachers were prompted to report any deviations of the script and reflect on their reasons to deviate. Additionally, teachers were asked “Did you feel your students understood all concepts?” (to be rated on a scale from 1-didn’t understand, 2-understood little, 3-understood ok, 4-fully understood, to 5-fully understood and capable of using new competencies) and “Do you feel the contents of this session are relevant for your students?” (to be rated on a scale from 1-not relevant, 2-little relevance, 3-ok, 4-relevant, to 5-highly relevant).

Table 1 lists all intervention group teachers and their number of completed sessions, completed fidelity questionnaires and average duration of their sessions. Average time spent on programme sessions is further detailed in table 2 across all sessions and all teachers. Overall average ratings across all sessions and teachers are provided in table 3. Teachers’ individual fidelity scores concerning content coverage, participant responsiveness and perceived relevance are reported in table 4. Respective scores per session are reported in table 5.

Regarding adherence to the programme, the number of completed sessions was used as main indicator and teachers were grouped into four implementation progress groups based on this. Only the “full programme” group (i.e. completed all eight sessions) was included in the main analysis. The additional adherence factors, duration of each session and programme coverage can be found in tables 1 and 4. On average, teachers who finished all programme sessions spent 59.2 minutes on each session, while teachers who partly finished the programme spent an average of 65.3 minutes on their sessions (see table 1). Similarly, teachers, who partly finished the programme, reported a slightly higher content coverage (M=3.22, SD=0.30) compared to teachers who finished the full programme (M=2.95, SD=0.19). For teachers who dropped out or finished few sessions only, not enough data is available to calculate a mean score.

To assess a possible association between teacher fidelity reports and students’ perceived teacher generated class climate after the intervention (T2) (i.e. did students feel the teacher actively encouraged a positive class climate), spearman correlation were computed (for raw data see table 6). There correlation between students’ perceived teacher class climate and teacher’s perceived relevance of contents was negligible (*r* = -.06, *p*= .83). There was a weak, negative correlation between students’ perceived teacher class climate and teacher’s reported student responsiveness (*r* = -.11, *p*= .71). There was a moderate, negative correlation between students’ perceived teacher class climate and teacher’s reported content coverage (*r* = -.50, *p*= .069). Content coverage was included in this analysis for the purpose of comprehensive reporting, however interpretation of any analysis using content coverage alone is difficult. As some teachers did not implement all eight sessions, implementation progress (i.e. how many sessions were implemented) was used as main indicator of adherence and implementation progress might be low even if content coverage for singular completed session might be high. Thus, content coverage alone is an incomplete indicator of adherence and analyses using content coverage alone are potentially misleading.

**Table 1**

*List of Intervention Group Teachers and Number and Duration of their Completed Sessions*

|  | Number of Competed Sessions | Progress Implementation group | Number of Fidelity Questionnaires Completed | Mean Session Duration |
| --- | --- | --- | --- | --- |
| Teacher 1 | 6 | partly finished | 5 | 60 |
| Teacher 2 | 2 | few session | 2 | 50 |
| Teacher 3 | 7 | partly finished | 1 | 100 |
| Teacher 4 | 7 | partly finished | 4 | 50 |
| Teacher 5 | N/A | drop out | N/A | N/A |
| Teacher 6 | 8 | full programme | N/A | N/A |
| Teacher 7 | 8 | full programme | N/A | N/A |
| Teacher 8 | 8 | full programme | 3 | 50 |
| Teacher 9 | 8 | full programme | 2 | 50 |
| Teacher 10 | 8 | full programme | 2 | 75 |
| Teacher 11 | 7 | partly finished | 7 | 64 |
| Teacher 12 | 8 | full programme | 3 | 47 |
| Teacher 13 | N/A | drop out | 4 | 43 |
| Teacher 14 | 8 | full programme | 5 | 50 |
| Teacher 15 | 7 | partly finished | 5 | 51 |
| Teacher 16 | 6 | partly finished | 4 | 68 |
| Teacher 17 | 8 | full programme | 8 | 84 |
| Teacher 18 | 2 | few session | N/A | N/A |
| Teacher 19 | N/A | drop out | N/A | N/A |

*Note.* This table lists all teachers of the intervention group and which programme progress group they are belong to, their number of completed fidelity questionnaires and their reported average time spend on sessions. N/A = not applicable.

**Table 2**

*Average Time Spent on One Programme Session*

| Session Duration in Minutes | | Frequency | | Percent | | Valid Percent | | Cumulative Percent | |  |
| --- | --- | --- | --- | --- | --- | --- | --- | --- | --- | --- |
|  | 25 | | 1 | | 1.7 | | 1.8 | | 1.8 | |
|  | 35 | | 1 | | 1.7 | | 1.8 | | 3.5 | |
|  | 40 | | 1 | | 1.7 | | 1.8 | | 5.3 | |
|  | 45 | | 6 | | 10.3 | | 10.5 | | 15.8 | |
|  | 50 | | 28 | | 48.3 | | 49.1 | | 64.9 | |
|  | 60 | | 2 | | 3.4 | | 3.5 | | 68.4 | |
|  | 70 | | 4 | | 6.9 | | 7.0 | | 75.4 | |
|  | 80 | | 8 | | 13.8 | | 14.0 | | 89.5 | |
|  | 90 | | 1 | | 1.7 | | 1.8 | | 91.2 | |
|  | 100 | | 5 | | 8.6 | | 8.8 | | 100.0 | |
|  | Total | | 57 | | 98.3 | | 100.0 | |  | |
|  | Missing | | 1 | | 1.7 | |  | |  | |
| Total | | 58 | | 100.0 | |  | |  | |  |

*Note.* This table shows how much time teachers spend on average on programme sessions.

**Table 3**

*Overview Fidelity Measures*

|  | N | Minimum | Maximum | Mean | SD |
| --- | --- | --- | --- | --- | --- |
| Content Coverage | 57 | 1.50 | 4.75 | 3.05 | 0.61 |
| Participant Responsiveness | 56 | 2.67 | 5.00 | 3.91 | 0.63 |
| Perceived Relevance | 57 | 3.00 | 5.00 | 4.12 | 0.45 |

*Note.* This table provides all three average fidelity score across all sessions and teachers.

**Table 4**

*Fidelity Results per Teacher*

|  | Content Coverage | | Participant Responsiveness | | Perceived Relevance | |
| --- | --- | --- | --- | --- | --- | --- |
|  | Mean | SD | Mean | SD | Mean | SD |
| Teacher 1 | 3.47 | 0.76 | 4.33 | 0.74 | 3.88 | 0.69 |
| Teacher 2 | 2.63 | 0.53 | 4.15 | 0.49 | 4.70 | 0.42 |
| Teacher 3 | 3.50 | N/A | 3.75 | N/A | 4.50 | N/A |
| Teacher 4 | 2.71 | 0.70 | 4.29 | 0.67 | 3.77 | 0.31 |
| Teacher 8 | 2.97 | 0.21 | 4.47 | 0.06 | 4.11 | 0.32 |
| Teacher 9 | 3.17 | 0.47 | 3.83 | 0.60 | 3.79 | 0.06 |
| Teacher 10 | 2.68 | 1.31 | 3.20 | 0.28 | 4.33 | 0.11 |
| Teacher 11 | 3.34 | 0.39 | 4.12 | 0.66 | 3.84 | 0.46 |
| Teacher 12 | 3.13 | 0.23 | 3.57 | 0.40 | 4.18 | 0.38 |
| Teacher 13 | 2.98 | 0.67 | 3.93 | 0.52 | 4.28 | 0.71 |
| Teacher 14 | 2.78 | 0.35 | 4.22 | 0.75 | 4.36 | 0.33 |
| Teacher 15 | 3.01 | 1.19 | 3.35 | 0.47 | 4.43 | 0.24 |
| Teacher 16 | 3.28 | 0.61 | 3.70 | 0.24 | 4.30 | 0.43 |
| Teacher 17 | 2.99 | 0.37 | 3.44 | 0.43 | 3.94 | 0.30 |

*Note.* This table provides teachers’ average self-reported fidelity scores across all their completed fidelity checks (only teacher 14 completed all 8 fidelity checklists). Number of teachers is congruent with teachers in table 1, teachers who did not complete any fidelity questionnaires have not been included in this table. N/A = not applicable.

**Table 5**

*Fidelity Results for each Programme Session*

|  | Content Coverage | | Participant Responsiveness | | Perceived Relevance | |
| --- | --- | --- | --- | --- | --- | --- |
|  | Mean | SD | Mean | SD | Mean | SD |
|  |  |  |  |  |  |  |
| Session 1 | 3.14 | 0.79 | 4.08 | 0.82 | 4.14 | 0.50 |
| Session 2 | 3.22 | 0.70 | 4.04 | 0.57 | 4.35 | 0.42 |
| Session 3 | 3.11 | 0.32 | 3.90 | 0.73 | 4.01 | 0.47 |
| Session 4 | 3.03 | 0.41 | 4.01 | 0.45 | 3.97 | 0.55 |
| Session 5 | 2.84 | 0.62 | 3.63 | 0.58 | 3.97 | 0.42 |
| Session 6 | 3.08 | 0.38 | 3.61 | 0.67 | 4.14 | 0.47 |
| Session 7 | 2.67 | 0.58 | 3.80 | 0.35 | 4.20 | 0.20 |
| Session 8 | 2.93 | 0.64 | 3.67 | 0.42 | 4.27 | 0.42 |

*Note.* This table provides average fidelity measures completed by teachers for each session.

**Table 6**

*Students’ Perceived Class Climate Generated by Teachers and Students for each Teacher*

|  | Teacher Climate T2 | | Student Class Climate T2 | |
| --- | --- | --- | --- | --- |
|  | Mean | SD | Mean | SD |
| Teacher 1 | 31.52 | 4.51 | 24.09 | 5.67 |
| Teacher 2 | 31.43 | 4.70 | 20.62 | 6.49 |
| Teacher 3 | 31.08 | 6.80 | 21.77 | 8.80 |
| Teacher 4 | 33.69 | 1.84 | 30.31 | 4.33 |
| Teacher 5 | 31.82 | 3.58 | 27.73 | 5.91 |
| Teacher 6 | 31.82 | 3.58 | 27.73 | 5.91 |
| Teacher 7 | 30.43 | 5.36 | 23.65 | 5.82 |
| Teacher 8 | 32.35 | 3.49 | 29.73 | 4.17 |
| Teacher 9 | 30.57 | 4.41 | 25.33 | 5.57 |
| Teacher 10 | 33.05 | 3.02 | 23.50 | 5.21 |
| Teacher 11 | 31.59 | 3.72 | 23.04 | 5.87 |
| Teacher 12 | 33.29 | 1.86 | 25.41 | 4.32 |
| Teacher 13 | 33.00 | 3.71 | 28.42 | 4.21 |
| Teacher 14 | 33.00 | 3.71 | 28.42 | 4.21 |
| Teacher 15 | 33.32 | 3.46 | 25.32 | 7.10 |
| Teacher 16 | 30.00 | 5.60 | 22.13 | 5.54 |
| Teacher 17 | 32.20 | 3.52 | 27.28 | 4.89 |

*Note.* Number of teachers is congruent with teachers in table 1 and 4. These data was collected after the intervention period (T2).

|  | Teacher Climate T2 | | Student Class Climate T2 | |
| --- | --- | --- | --- | --- |
|  | *ρ* | *p* | *ρ* | *p* |
| Content Coverage | -.50 | .069 | -.35 | .214 |
| Participant Responsiveness | -.11 | .708 | .32 | .263 |
| Perceived Relevance | -06 | .834 | -.49 | .076 |

**Table 7**

*Correlations Between Fidelity Measures and Class Climate Measures*

*Note. S*pearman’s correlation coefficient *ρ* and *p*-values to assess the correlation’s statistical significance were computed.
